# Insight into resistance to ‘*Candidatus* Liberibacter asiaticus,’ associated with Huanglongbing, in Oceanian citrus genotypes

**DOI:** 10.3389/fpls.2022.1009350

**Published:** 2022-09-09

**Authors:** Mônica N. Alves, Laudecir L. Raiol-Junior, Eduardo A. Girardi, Maéva Miranda, Nelson A. Wulff, Everton V. Carvalho, Sílvio A. Lopes, Jesus A. Ferro, Patrick Ollitrault, Leandro Peña

**Affiliations:** ^1^Fundo de Defesa da Citricultura, Araraquara, Brazil; ^2^Faculdade de Ciências Agrárias e Veterinárias (FCAV), Universidade Estadual Paulista (UNESP), Jaboticabal, Brazil; ^3^Empresa Brasileira de Pesquisa Agropecuária, Cruz das Almas, Brazil; ^4^CIRAD, UMR AGAP Institut, Montpellier, France; ^5^AGAP Institut, Univ. Montpellier, CIRAD, INRAE, Institut Agro, Montpellier, France; ^6^Instituto de Biologia Molecular y Celular de Plantas – Consejo Superior de Investigaciones Científicas, Universidad Politécnica de Valencia, Valencia, Spain

**Keywords:** greening, HLB, Rutaceae, Aurantioideae, *Microcitrus*, *Eremocitrus*, citrus breeding

## Abstract

Huanglongbing (HLB), the most destructive citrus disease, is associated with unculturable, phloem-limited *Candidatus* Liberibacter species, mainly *Ca.* L. asiaticus (Las). Las is transmitted naturally by the insect *Diaphorina citri*. In a previous study, we determined that the Oceanian citrus relatives *Eremocitrus glauca*, *Microcitrus warburgiana*, *Microcitrus papuana*, and *Microcitrus australis* and three hybrids among them and *Citrus* were full-resistant to Las. After 2 years of evaluations, leaves of those seven genotypes remained Las-free even with their susceptible rootstock being infected. However, Las was detected in their stem bark above the scion-rootstock graft union. Aiming to gain an understanding of the full-resistance phenotype, new experiments were carried out with the challenge-inoculated Oceanian citrus genotypes through which we evaluated: (1) Las acquisition by *D. citri* fed onto them; (2) Las infection in sweet orange plants grafted with bark or budwood from them; (3) Las infection in sweet orange plants top-grafted onto them; (4) Las infection in new shoots from rooted plants of them; and (5) Las infection in new shoots of them after drastic back-pruning. Overall, results showed that insects that fed on plants from the Oceanian citrus genotypes, their canopies, new flushes, and leaves from rooted cuttings evaluated remained quantitative real-time polymerase chain reaction (qPCR)-negative. Moreover, their budwood pieces were unable to infect sweet orange through grafting. Furthermore, sweet orange control leaves resulted infected when insects fed onto them and graft-receptor susceptible plants. Genomic and morphological analysis of the Oceanian genotypes corroborated that *E. glauca* and *M. warburgiana* are pure species while our *M. australis* accession is an *M. australis* × *M. inodora* hybrid and *M. papuana is* probably a *M. papuana* × *M. warburgiana* hybrid. *E. glauca* × *C. sinensis* hybrid was found coming from a cross between *E. glauca* and mandarin or tangor. *Eremocitrus* × *Microcitrus* hybrid is a complex admixture of *M. australasica*, *M. australis*, and *E. glauca* while the last hybrid is an *M. australasica* × *M. australis* admixture. Confirmation of consistent full resistance in these genotypes with proper validation of their genomic parentages is essential to map properly genomic regions for breeding programs aimed to generate new *Citrus*-like cultivars yielding immunity to HLB.

## Introduction

Huanglongbing (HLB), also known as citrus greening, is a ravaging citrus disease that has been concerning citrus growers and researchers across the world, especially during the last two decades, when it spread from South East Asia to West Asia, North Oceania, America, and several African countries (Alquézar et al., 2022). HLB is associated with the presence of the unculturable, Gram-negative, and phloem-limited α-proteobacteria ‘*Candidatus* Liberibacter africanus’ (Laf), ‘*Ca*. L. asiaticus’ (Las) and ‘*Ca.* L. americanus’ (*C*Lam) (Bové, 2006), being Las the most prevalent and aggressive species (Bassanezi et al., 2020). In the field, Las is transmitted by the insect vector *Diaphorina citri* (Kuwayama) (Hemiptera: Liviidae), commonly known as the Asian citrus psyllid (ACP; Capoor et al., 1967). In Brazil, HLB management relies on the implementation on an area-wide basis of the three-pronged system (TPS) which consists of the elimination of symptomatic trees, use of certified healthy nursery trees for new plantings and resets, and intensive insecticide control of *D. citri* (Bassanezi et al., 2020). This strategy has been executed since the first description of HLB in Brazil and mainly due to these practices, HLB has only reached 22% of the trees in the citriculture belt of São Paulo State and Triângulo Mineiro (Fundecitrus, 2021), a scenario that would be worst without the implementation of the TPS. However, this strategy has not been adopted and maintained by all growers, ACP resistance to insecticides is arising and thus HLB incidence has gradually increased, demanding new approaches to cope with the disease.

Leaves from HLB-affected trees usually show asymmetrical chlorosis or blotchy mottle, and, at later stages of infection, thicker and enlarged veins (Bové, 2006). Fruits are smaller and misshapen while the juice is highly acidic and poor in soluble solids (Bassanezi et al., 2009). Symptomatic leaves and fruits drop prematurely (Moreira et al., 2022). Leaf symptoms can be confusing, especially at the early stages of infection, and, thus, not always can be used for diagnosis purposes (Gottwald et al., 2007). The quantitative real-time polymerase chain reaction (qPCR) was then developed for Las, becoming the most important and widely used tool for HLB diagnosis and research (Li et al., 2006). However, qPCR does not distinguish the DNA of dead from live bacteria while DNA from dead bacteria is only slowly degraded in plant tissues (Josephson et al., 1993). This can pose problems making the interpretation of results of some works difficult, especially for bacterial titers close to detection limits.

Evaluation by qPCR of citrus genotypes challenge-inoculated with Las in controlled and field conditions has shown that all commercial cultivars are susceptible (Folimonova et al., 2009; Miles et al., 2017). However, some citrus relatives from other genera within the Aurantioideae subfamily have been recently reported as completely or partially resistant to HLB (Ramadugu et al., 2016; Alves et al., 2021a,b). This includes the Australian desert lime, *Eremocitrus glauca* (Lindl.) Swingle, and several Oceanian *Microcitrus* species. Both genera belong to the true-citrus fruit trees group, are monoembryonic, and cross-compatible with *Citrus* (Swingle and Reece, 1967). Furthermore, *E. glauca*, some *Microcitrus* species, and their hybrids with *Citrus* have shown to be suitable citrus rootstocks (Bitters et al., 1964, 1977) and presented good graft compatibility with the rootstock ‘Rangpur’ lime (*Citrus* × *limonia* Osbeck) when used as scions (Alves et al., 2021b).

In field experiments, seedlings of *E. glauca* and some *Microcitrus* genotypes were identified as potential sources of resistance or tolerance to HLB (Ramadugu et al., 2016). In controlled greenhouse experiments, by using clonal plants grafted onto the Las-susceptible ‘Rangpur’ lime rootstock and upon challenging by graft-inoculation of Las-infected budwood on both the scion and rootstock, Alves et al. (2021b) discerned seven full-resistant citrus genotypes, namely, *E. glauca*, *M. australis*, *M. warburgiana*, and *M. papuana* and three hybrids among them and with sweet orange within a collection of Citreae species. Although the susceptible rootstock became infected with Las, the bacterium was never detected in the scion leaves of these genotypes thus becoming the most promising germplasm among Oceanian citrus to be used as parents in breeding efforts for generating citrus-like cultivars resistant to Las (Alves et al., 2021b). Because of this, the introgression of HLB resistance into cultivated citrus has become tangible in citrus breeding programs (Alquézar et al., 2021). However, Las was found in the stem bark sampled at 5 cm above the scion-rootstock union of all the seven full-resistant genotypes and unevenly detected at 30 cm height in two of them (Alves et al., 2021b). Las amplifications at these different distances from the infected rootstock led to queries on whether the detected bacterial DNA was viable. Aiming to better characterize the resistance of those genotypes and further understand Las movement within the stem bark of the grafted plant materials, a set of five experiments was carried out. In addition, to support interpretation of the Las resistance phenotype, the genetic background of the seven Oceanian citrus genotypes was assessed by genotyping by sequencing (GBS) analysis.

## Materials and methods

### Phenotyping of Oceanian citrus genotypes

Morphological analysis of the Oceanian citrus putatively true species was performed with 4–14 healthy plants per genotype propagated onto the ‘Rangpur’ lime rootstock. The aspect of leaves, spines, flowers, and fruits was compared with their description made by Swingle and Reece (1967), also considering the results obtained from their genome analysis, to ascertain the presumed pure or hybrid nature of each genotype.

### High-density SNP genotyping of Oceanian citrus genotypes

Aiming to genetically characterize the Fundecitrus collection used for HLB resistance studies (Alves et al., 2021b), a GBS analysis was performed with the seven genotypes described as full-resistant by Alves et al. (2021b) [*M. warburgiana* (F.M. Bailey) Tanaka, *M. papuana* Winters (revealed to be actually an *M. papuana* hybrid in the present work), *M. australis* (A. Cunn. ex Mudie) Swingle (actually an *M. australis* hybrid), *E. glauca* (Lindl.) Swingle, a *Microcitrus* sp. hybrid (formerly called *Microcitrus* sp. × *E. glauca*), an *E. glauca* × *Microcitrus* sp. complex hybrid, and a supposed *E. glauca* × *Citrus* × *sinensis* hybrid (eremorange)], and the susceptible sweet orange cultivar ‘Tobias’ [*C*. × *sinensis* (L.) Osbeck]. Two finger lime (*M. australasica*) accessions of the Fundecitrus germplasm (‘True Sanguinea’ and a ‘Green’ one) considered as representative of *M. australasica* as well as one accession of each *Clymenia polyandra*, *M. inodora*, and *M. garrawayae* were included to check their status of pure species representatives and the potential contribution of the different species to the selected resistant germplasm. Five varieties from the CRB-Citrus collection, San Giuliano (Corsica) whose phylogenomic constitution was previously analyzed by GBS or WGS (Wu et al., 2014, 2018; Ahmed et al., 2019), were also added to the GBS analysis as a reference to check the quality of the results: Nules clementine ICVN 0100389, Eureka lemon ICVN 0100289, Star Ruby grapefruit ICVN 0100293, eremorange SRA871, and Australian finger lime SRA1002. In addition, published WGS data available in NCBI (Wang et al., 2017; Wu et al., 2018) were used to mine diagnostic SNPs (DSNPs) of ancestral taxa from pure representative accessions: *C. maxima* (SRR5128240 and SRR3824065), *C. medica* (SRR3938056 and SRR3944139), *C. reticulata* (SRR3747399 and SRR3747527), *M. australis* (SRR5128218 and SRR6188449), and *M. australasica* (SRR5128220).

For GBS analysis, the genomic DNA was isolated using the Plant DNAeasy kit (Qiagen) according to the manufacturer’s instructions. The genomic DNA concentration of each sample was adjusted to 20 ng/μL, and ApekI GBS libraries were prepared following the protocol described by Elshire et al. (2011) with 96 DNA samples multiplexed per GBS library (the accessions used for this paper were included in another larger experiment). Ten μL of each DNA sample (200 ng) was digested with the ApekI enzyme (New England Biolabs, Hitchin, United Kingdom). Digestion took place at 75°C for 2 h and then at 65°C for 20 min for enzyme inactivation. The ligation reaction was completed in the same plate as the digestion, using the T4 DNA ligase enzyme (New England Biolabs, Hitchin, United Kingdom) at 22°C for 1 h. Then, the ligase was inactivated prior to pooling the samples by holding it at 65°C for 20 min. For each library, ligated samples were pooled and PCR-amplified in a single tube. Genome complexity was reduced using PCR primers with one selective base (A) as described by Sonah et al. (2013). Pair-end sequencing was performed on one lane of an Illumina HiSeq4000 platform at Genewiz facilities. RAW sequencing data were cleaned with cutadapt (Martin, 2011) and demultiplexed with GBSX (Herten et al., 2015). Clean demultiplexed sequencing data from GBS are available in the NCBI SRA (Sequence Read Archive), under accession number (Submission ID: PRJNA862483). SNP genotype calling was performed with the GATK4 pipeline (Van der Auwera and O’Connor, 2020). The reference genome was constituted by the nuclear scaffolds of the Clementine v1.0 genome assembly^1^, the mitochondrial (Yu et al., 2018), and chloroplast (Bausher et al., 2006) reference genomes from *C.* × *sinensis* (available in NCBI with reference NC_037463 and NC_008334, respectively). Positions with less than 10 reads were considered as missing data. Polymorphic positions were filtered for diallelic SNPs and minor allele frequency greater than 0.01.

### Genetic data analysis

Neighbor-joining analyses for nuclear and mitochondrial data as well as a factorial analysis for nuclear data were performed to describe the genetic diversity organization of the analyzed germplasm. Both analyses are based on a dissimilarity matrix established from SNP genotyping data using the Manhattan dissimilarity index:

$$D_{\mathrm{ij}}=\frac{1}{K}\,\sum_{1}^{K}\left|x_{\mathrm{ik}}-x_{\mathrm{jk}}\right|$$

where i and j are the two individuals, k is the locus, K is the total number of loci, and xik is the frequency of the alternative allele at locus k for the individual i. All analyses were performed with DARwin software version 6.0^2^.

Ancestral genome contribution along the genome of the admixed genotype was analyzed based on the distribution of homozygosity and heterozygosity of ancestral diagnostic SNPs (DSNPs). Fourteen accessions were used to select diagnostic SNPs for 10 ancestral species: two accessions for *C. maxima*, *C. reticulata*, *C. medica*, and *M. australis* and one for *M. australasica*, *M. inodora*, *M. warburgiana*, *M. garrawayae*, *E. glauca*, and *Clymenia polyandra*. For each ancestor, DSNPs that fully differentiated the considered ancestor from all other species (i.e., all accessions of the considered ancestor were homozygous for the same allele and all other accessions homozygous for the second allele) were filtered in Excel. For each ancestor, homozygosity and heterozygosity curves along the genome were established with sliding windows of 20 consecutive DSNPs for the considered ancestor (i.e., the proportion of the 20 markers in homozygosity or heterozygosity for the alleles of the considered ancestor). The medium position of the 20 markers of each sliding window was used to draw the curve in Circos representation (Krzywinski et al., 2009) of the true Galaxy interface (Rasche and Hiltemann, 2020).

### Plant materials and growth conditions for Las resistance assessment

**FIGURE 1:**
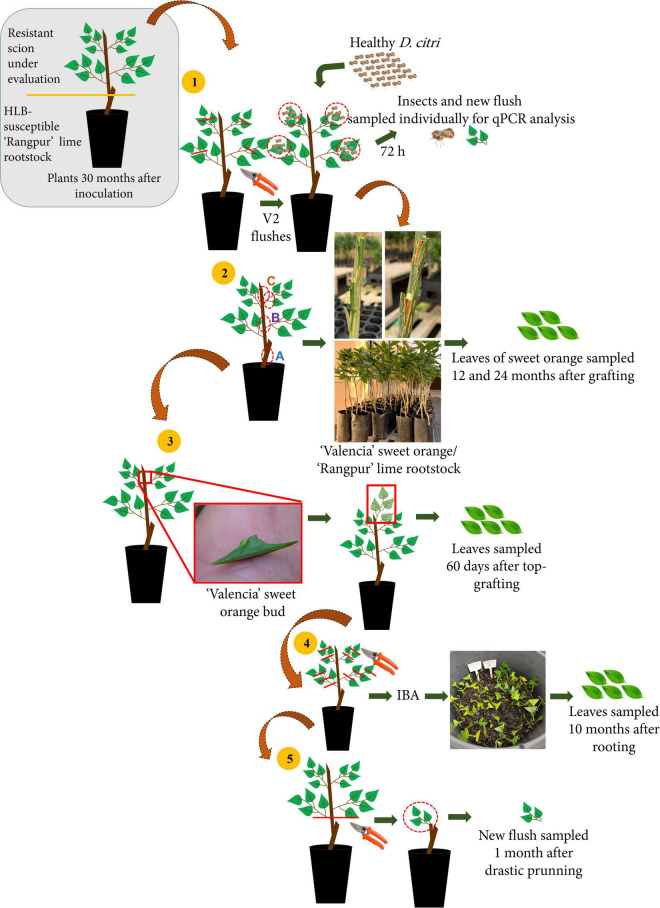
Schematic design of the five experiments carried out **(1)** Las acquisition by *Diaphorina citri* reared on Oceanian citrus genotypes; **(2)** Las indexing in ‘Valencia’ sweet orange plants grafted with (A) bark tissue from 5 cm below the graft union of Las-infected ‘Rangpur’ lime used as rootstock, (B) bark tissue from 8 cm above the graft union, and (C) budwood from the top of the Oceanian citrus genotypes; **(3)** Top-grafting of ‘Valencia’ sweet orange plants on scions from the Oceanian citrus genotypes; **(4)** Las multiplication through stem cuttings of Oceanian citrus genotypes; and **(5)** Forcing Las colonization from the ‘Rangpur’ lime rootstock to new sprouted shoots of Oceanian citrus genotypes after severe back-pruning.

In this work, the plants of the Oceanian citrus genotypes were the same used in the previous work by Alves et al. (2021b) and included *M. warburgiana* (F.M. Bailey) Tanaka, *M. papuana* Winters (revealed to be actually a *M. papuana* hybrid in the present work), *M. australis* (A. Cunn. ex Mudie) Swingle (actually a *M. australis* hybrid), *E. glauca* (Lindl.) Swingle, a *Microcitrus* sp. hybrid (formerly called *Microcitrus* sp. × *E. glauca*), an *E. glauca* × *Microcitrus* sp. complex hybrid, and a supposed *E. glauca* × *Citrus* × *sinensis* hybrid (eremorange), being the susceptible sweet orange cultivar ‘Tobias’ [*C*. × *sinensis* (L.) Osbeck] used as control. All these accessions were propagated onto Las-susceptible ‘Rangpur’ lime nucellar rootstocks. Budwood used to propagate each accession came from a single donor mother tree. Plants were kept in a greenhouse with air temperature varying from 20.0 to 30.0°C and natural sunlight conditions. They were grown in 4 L polyethylene bags filled with coir, fertigated twice a week, and sprayed with insecticides and miticides monthly. Other information on the accessions, challenge-inoculation procedures, and plant maintenance practices are described in detail in Alves et al. (2021b). Oceanian citrus and ‘Tobias’ sweet orange control plants used for experiments 1 to 5 reported here (Figure 1) were ∼ 3 to 4-year-old. Las had been detected in all rootstock bark samples by qPCR with an average cycle threshold (Ct) of 30.0 ± 0.4, and in the ‘Tobias’ sweet orange control scion leaves (Ct 23.8 ± 1.4), at 30 months after graft inoculation. Some composite plants from all the seven Oceanian citrus and ‘Tobias’ sweet orange challenge-inoculated genotypes died along the course of the experiments due to the damage caused by Las infection to their ‘Rangpur’ lime root system.

### Experiment 1: Las acquisition by *Diaphorina citri* reared on Oceanian citrus genotypes

Thirty months after Las graft challenge-inoculation and the categorization of the seven Oceanian citrus accessions as resistant, the tip of the branches from the canopy of those plants that remained alive was pruned to promote flushing. Three to four flushes per plant in the V2 phenological stage of development (Cifuentes-Arenas et al., 2018) were selected and used to confine five 20-day-old Las-free *D. citri* adults per flush for 72 h of the acquisition access period (Figure 1.1). The insects were obtained from a colony that had been reared on Las-free *Murraya paniculata* (L.) Jack seedlings. After the confinement, insects and 5–10 leaves of each flush were sampled individually, DNA was extracted and Las detection was assessed by qPCR. As a control, Las-infected ‘Tobias’ sweet orange plants were used.

### Experiment 2: Las infection in ‘Valencia’ sweet orange plants grafted with bark and budwood from the Oceanian citrus genotypes

**FIGURE 2:**
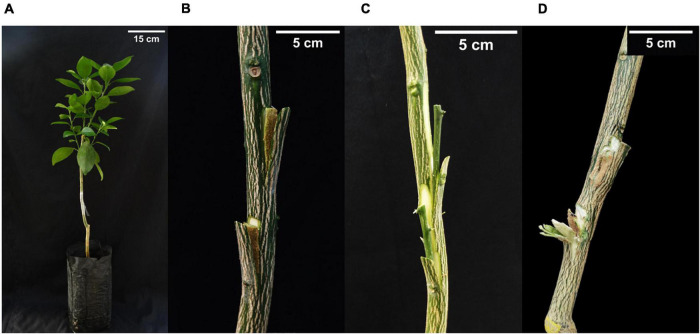
Representative aspect **(A)** of a ‘Valencia’ sweet orange plant grafted with bark or budwood from the Oceanian citrus genotypes in Experiment 2; **(B)** of bark tissue taken from 5 cm below the graft union of Las-infected ‘Rangpur’ lime used as rootstock or from 8 cm above the graft union; **(C)** of budwood removed from the top of the Oceanian citrus genotypes; **(D)** and from a failed bark graft in experiment 2.

About 174 2-year-old plants of ‘Valencia’ sweet orange on ‘Rangpur’ lime rootstock were grafted with bark patches and budwood from the seven Oceanian citrus genotypes and ‘Tobias’ sweet orange as a control to assess the eventual transmission of Las from bark and budwood pieces of challenge-inoculated plants to healthy ‘Valencia’ sweet orange plants (Figures 1.2, 2A). All ‘Valencia’ sweet orange plants were grown in 4 L containers. Two patches of bark tissue (Figure 2B) or budwood (Figure 2C) with ca. 3–5 cm long by 0.8 cm wide size from 4 to 11 individual plants of each Oceanian citrus genotype were grafted at 15 cm above the scion-rootstock union of each ‘Valencia’ sweet orange tree. The bark and budwood tissues were collected from three different regions of each plant from the Oceanian citrus genotypes evaluated: at 5 cm below the scion-rootstock union (‘Rangpur’ lime trunk) and 8 cm above it (Oceanian citrus genotype trunk) and at the highest shoot of the scion canopy of all available plants (budwood). As a control, the same approach was used with bark and budwood tissue from Las-infected ‘Tobias’ sweet orange plants. A sample of 0.2 g from each bark and budwood piece to be grafted in ‘Valencia’ sweet orange plants was used to assess Las by qPCR analysis. Three months after grafting, the ‘Valencia’ sweet orange plants were top pruned at about 1.5 cm distant from the apex to stimulate the development of new shoots and, consequently, to force Las movement from the bark and budwood tissue used as the eventual inoculum source to the new tissues of ‘Valencia’ sweet orange (Raiol-Junior et al., 2021a). The union of the bark and budwood tissue with the ‘Valencia’ sweet orange stems was visually observed to evaluate the graft success (Figure 2D). After 12 and 24 months of grafting, four to six leaves were randomly sampled in the ‘Valencia’ sweet orange scion of all tested plants and processed for DNA extraction and qPCR analysis for Las detection.

### Experiment 3: Top-grafting of ‘Valencia’ sweet orange plants on scions from the Oceanian citrus genotypes

Buds from a Las-free greenhouse-grown ‘Valencia’ sweet orange plant were collected and grafted onto the highest point of the scion top of 4–11 plants from the seven Oceanian citrus genotypes and ‘Tobias’ sweet orange, used as a control (Figure 1.3). This experiment aimed to determine whether Las would be able to translocate from the infected ‘Rangpur’ lime rootstock through the Oceanian citrus genotypes, now used as interstock and colonize the top-grafted ‘Valencia’ sweet orange scions. Sixty days after the ‘Valencia’ buds had grown and developed as scions, their leaves were randomly sampled for DNA extraction and qPCR analysis.

### Experiment 4: Las multiplication in cuttings of Oceanian citrus genotypes

To further assess whether the scion tissues of the Oceanian citrus genotypes were free of Las, stem cuttings were propagated for rooting (Figure 1.4). Twenty cuttings with 5-8 cm were collected from partially lignified shoots of the canopy from the Oceanian citrus genotypes *M. australis* hybrid, *E. glauca*, eremorange, *Microcitrus* sp. hybrid, and *E. glauca* × *Microcitrus* sp. hybrid and ‘Tobias’ sweet orange as a control. Two leaves were kept in each cutting top and they were cut in half to reduce transpiration. Cutting basal ends were immersed for 0.5 s in a rooting aqueous solution of synthetic auxin indole-3-butyric acid (IBA) (5,000 ppm), then they were disposed inside 12 L polyethylene plastic containers filled with decomposed pine bark substrate, (Terra do Paraíso, Holambra, São Paulo, Brazil) and were manually irrigated whenever necessary. The containers were maintained inside a wet chamber for 90 days. Ten months after cutting propagation, four to six leaves of each cutting-derived plant remaining alive were sampled for Las detection through qPCR.

### Experiment 5: Forcing Las colonization in shoots of Oceanian citrus genotypes

All original plants remaining alive from *M. australis* hybrid, *E. glauca*, eremorange, *Microcitrus* sp. hybrid, and *E. glauca* × *Microcitrus* sp. hybrid and ‘Tobias’ sweet orange as control were drastically back-pruned at 5 cm above the scion-rootstock union to force new flush growth and Las movement from the Las-infected ‘Rangpur’ lime rootstock to new leaves on the forced grown scion flush. Thirty days after pruning, 5–10 leaves of the newly emerged flushes per plant were randomly sampled for DNA extraction and qPCR analysis (Figure 1.5).

### Evaluation by quantitative real-time polymerase chain reaction

Samples were processed as described by Teixeira et al. (2005) using a TissueLyser II system (Qiagen, Valencia, CA, United States) at 45 Hz for 30 s in a 2.0-ml microtube containing 5-mm steel beads. For insect processing, one insect and a 3-mm bead were used in a 1.5 mL microtube. Total DNA were obtained from 0.5 g of leaf midribs, or 0.3 g of root and bark tissue, using a CTAB (cetyltrimethylammonium bromide) buffer (2% CTAB; 1.4 M NaCl; 2% PVP 10,000; 0.5 M EDTA pH 8; 1 M Tris–HCl pH 8; 0.2% b-mercaptoethanol) as mentioned in Murray and Thompson (1980). DNA was resuspended in Milli-Q^®^ water. DNA quality and concentration were measured using a nanodrop (Thermo Fisher Scientific, Massachusetts, EUA) (Desjardins and Conklin, 2010). For qPCR and detection of the 16S rDNA from Las, 1 μL of total DNA (100 ηg/μL), TaqMan^®^ PCR Master Mix (1×) (Invitrogen, Carlsbad, CA, United States), and Las-specific primers/probe (0.5 μM/0.2 μM) were used in a StepOnePlus thermocycler (Applied Biosystems, CA, United States), as described by Li et al. (2006). As an internal control for the quality of plants and psyllids DNA, the mitochondrial gene cytochrome oxidase (*COX*) (Li et al., 2006) and an insect wingless (*Wg*) (Manjunath et al., 2008) gene region were used, respectively. Furthermore, leaf and insect samples, negative and positive for Las, were used during DNA extractions and qPCR assays as negative and positive controls, respectively. qPCR cycle threshold (*C*t) values higher than 34.0 produce variable results (Lopes et al., 2013); therefore, samples were classified as ‘positive’ with *C*t ≤ 34.0, suspected to be positive with 34.0 < *C*t values < 36.0, and negative with *C*t > 36 and/or when non-detected. Average *C*t and the standard deviation values of treatments were calculated using positive *C*t values.

## Results

### Phenotyping analysis of Oceanian citrus genotypes

**FIGURE 3:**
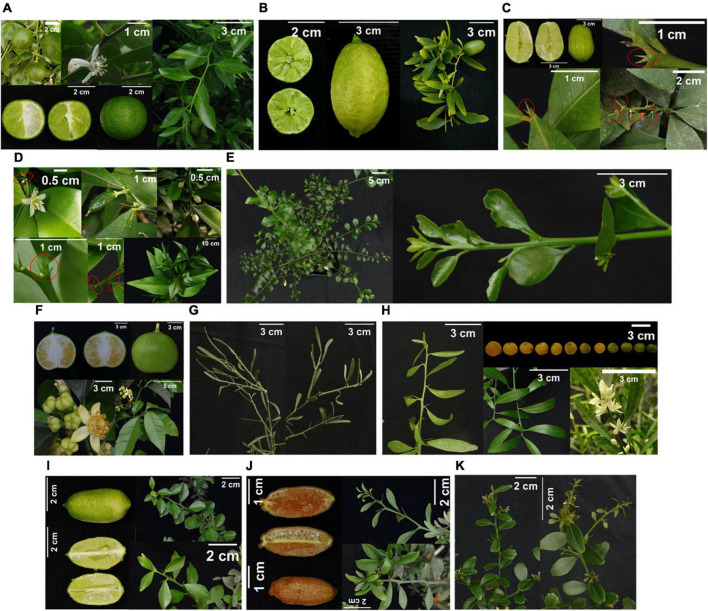
Morphological characterization of **(A)** *M. warburgiana*; **(B)** *M. papuana* × *M. warburgiana* hybrid; **(C)** *M. australis* × *M. inodora* hybrid; **(D)** *M. inodora*; **(E)** *M. garrawayae;* **(F)** *Clymenia polyandra;* **(G)** *E. glauca;* **(H)** *E. glauca* × *Citrus* sp. hybrid; **(I)** *M*. *australasica* × (*M. australis* × *M. australasica*) hybrid; **(J)** [*E. glauca* × (*M. australis* × *M. australasica*)] × *M. australasica* hybrid; and **(K)** *M. australasica.*

The *M. warburgiana* accession used in this study showed axillary solitary spines and elongate-elliptical leaves as well as small and globular fruit with six segments (Figure 3A), coincident with its description by Swingle and Reece (1967). The *M. papuana* accession used by us showed small and slightly elongated leaves and yielded semi-globose fruit (Figure 3B), which resembled a hybrid rather than a pure species. According to Winters (1976), *M. papuana* produces narrower, elongated leaves as well as long, cylindrical fruit.

The Australian round lime *M. australis* is easily distinguished from all the other *Microcitrus* species by its globose or slightly pear-shaped, rough-skinned fruit. Our *M. australis* genotype showed slightly pear-shaped fruit (Figure 3C), and instead solitary, paired spines were predominant in its leaf axils, which are characteristic of *M. inodora* (Figure 3D), suggesting that the *M. australis* hybrid used in this study may be an *M. australis* × *M. inodora* hybrid.

The phenotypic traits of *M. garrawayae* (Figure 3E) and *Clymenia polyandra* (Figure 3F) accessions from Fundecitrus also fit with the description of the species by Swingle and Reece (1967). The *M. garrawayae* accession presents small narrow, thick and leathery, lozenge-shaped to broadly lanceolate leaves, and the *C. polyandra* one has spineless twigs with leaves being very peculiar, with elliptical shape, acuminating at both ends, and with a wingless petiole. In addition, *C. polyandra* produces single flowers in the axils of the leaves.

*Eremocitrus* genus is one of the most distinctive among near *Citrus* relatives. Morphological traits were similar in our *E. glauca* accession (Figure 3G) and the one described by Swingle and Reece (1967). In general, the hybrids described between *Eremocitrus* and *Citrus* (Swingle and Reece, 1967) showed unifoliolate leaves of intermediate size between those of the two parental species, but thinner and bifacial, thus more similar to *Citrus* leaves, which coincided with the characteristics presented by the *E. glauca* × *C.* × *sinensis* accession used in this study (Figure 3H).

Regarding the other *Microcitrus* hybrids, *Microcitrus* sp. (Figure 3I) and *E. glauca* × *Microcitrus* sp. (Figure 3J) hybrids showed leaf, spines and fruit traits resembling the Australian finger lime *M. australasica* (Figure 3K). However, *Microcitrus* sp. fruit is subglobose, not fusiform as that of typical finger limes, suggesting that it may be a hybrid with another *Microcitrus* species yielding globose fruit as the Australian round lime. In the case of *E. glauca* × *Microcitrus* sp. hybrid, its leaves showed a narrow, thicker, coriaceous aspect resembling those of the eremorange but the fruits were long, cylindric, and slender, with red color, indicating that this genotype may be a hybrid between *E. glauca* and *M. australasica* (Figure 3I). Accumulation of anthocyanins providing red color to fruit is common in some *M. australasica* accessions.

### Genotyping analysis

After filtering the diallelic SNPs for less than 20% of missing data with the VCF tool, we obtained a genotyping matrix of 49187 diallelic SNPs for 27 accessions for the nuclear genome and a matrix with 40 SNPs for the mitochondrial genome. Only five SNPs were obtained for the chloroplast genome and were not found useful for maternal inheritance analysis.

**FIGURE 4:**
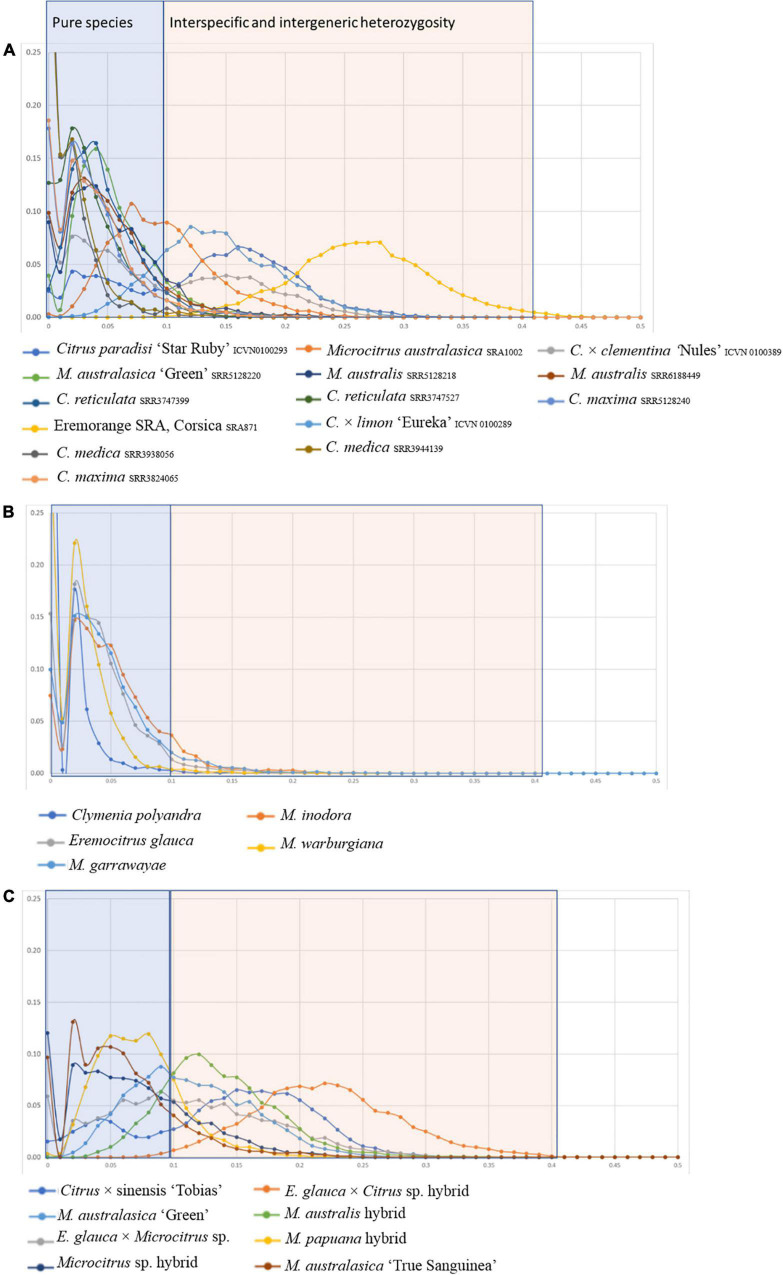
Distribution of heterozygosity values for the 27 accessions, established from sliding windows of 100 successive SNPs. **(A)** Already published genome with known phylogenomic status; **(B)** pure specific accessions from Fundecitrus Oceanian germplasm; **(C)** interspecific and admixed accessions of Fundecitrus Oceanian germplasm.

The analysis of heterozygosity performed for the Fundecitrus and Corsican accessions in comparison with already published accessions (Figure 4A) allowed to identify pure species representative with most of the heterozygosity distribution curve under 10% (Figure 4B) from admixed and interspecific hybrid accessions (Figure 4C).

Among the Fundecitrus germplasm *M. warburgiana*, *M. garrawayae*, *M. inodora*, *E. glauca*, and *C. polyandra* appear as pure representatives of their species while *M. australis*, *Eremocitrus* × *Microcitrus*, *Microcitrus* sp., *M. papuana*, and the three finger limes (the ‘True Sanguinea’ and the ‘Green’ accessions from Fundecitrus and the accession from Corsica) appeared to result from interspecific admixture. *E. glauca* × *C.* × *sinensis* from Fundecitrus displayed a very similar curve than one of the eremorange accessions of Corsica with a high value of heterozygosity resulting from the strong differentiation between *E. glauca* and the Asian *Citrus* species.

**FIGURE 5:**
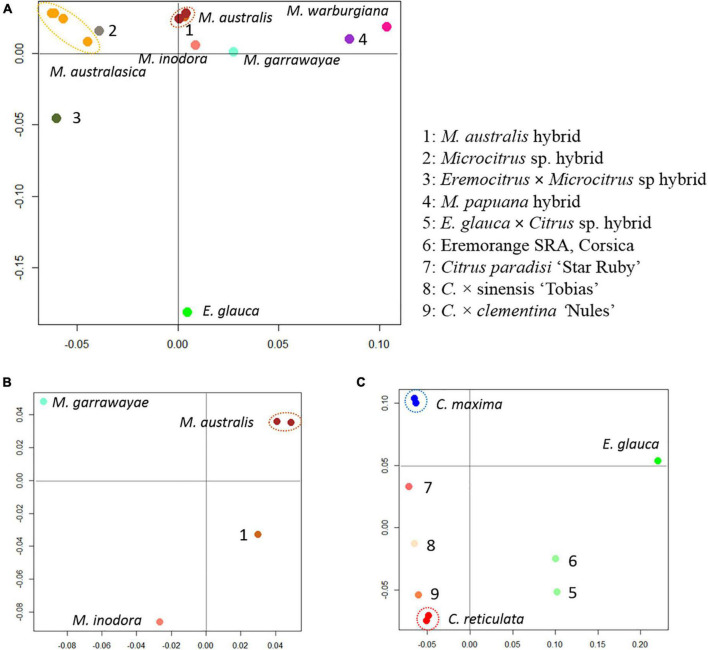
Factorial analysis from the genotyping of 49,187 diallelic SNPs. **(A)** Organization of the genetic diversity of the Oceanian germplasm. **(B)** Focus on the relationship of *M. australis* hybrid accession with *M. australis*, *M. garrawayae*, and *M. inodora*. **(C)** Focus on the relationship of the two supposed eremoranges with the *C. reticulata*/*C. maxima* gene pools.

**FIGURE 6:**
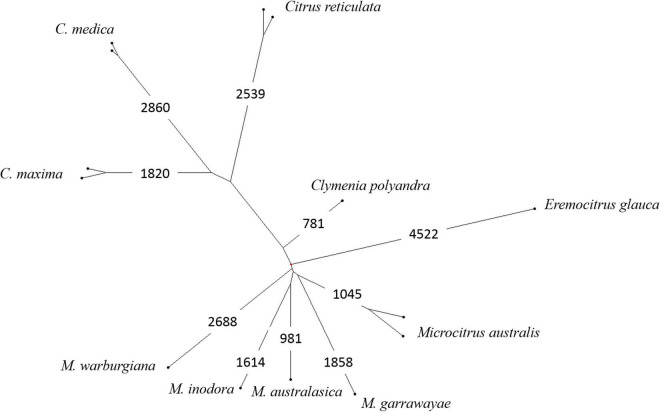
Organization of the nuclear diversity of 10 Asian and Oceanian species; NJ tree analysis from the genotyping of 49,187 diallelic SNPs and number of DSNPs for each species.

Factorial analyses of a subset of the data provided the first elements on the origin of the admixed accessions. A first factorial analysis focused in the Oceanian accessions (Figure 5A). The first axis (28.9%) opposes the finger limes accessions that are close to the true *M. australasica* representative and the species from Papua-New Guinea (*M. warburgiana* and *M. papuana*). The second axis (26.0%) discriminate the *E. glauca* accessions from the *Microcitrus* ones. The *Eremocitrus* × *Microcitrus* hybrid has an intermediate position in this axis confirming the intergeneric admixture and its position on the first axis suggests a major contribution of *M. australasica*. The position of *Microcitrus* sp. accession suggests no, or very low, *E. glauca* contribution and a major *M. australasica* one. *M. australis*, *M. inodora*, and *M. garrawayae* were still poorly resolved in this analysis and were subjected to an independent factorial analysis (Figure 5B). The first axis (48%) opposes *M. garrawayae* and *M. australis* pure representative accessions while the second axis discriminates *M. inodora* from all other pure species representatives. The intermediate position of the *M. australis* accession of Fundecitrus between the two true *M. australis* accessions and *M. inodora*, in addition to its important heterozygosity, suggests that it may be an interspecific F1 hybrid of *M. australis* × *M. inodora.* The last analysis (Figure 5C) concerned the supposed intergeneric hybrids between *Eremocitrus* and *C.* × *sinensis*, the representative accessions of the corresponding ancestors (*E. glauca, C. maxima*, and *C. reticulata*) as well as some admixed genotypes between these two citrus species. The first axis (61.1% of the total diversity) differentiates *E. glauca* from the *Citrus* species while axis 2 (26.9%) separates *C. maxima* from *C. reticulata* with clementine, sweet orange, and grapefruit in intermediate positions fully in agreement with their known phylogenomic constitution (Wu et al., 2014, 2018; Ahmed et al., 2019). The supposed intergeneric *E. glauca* × *C.* × *sinensis* accession from Fundecitrus is close to the eremorange from Corsica. On axis 1, these two accessions have an intermediate position between *Eremocitrus* and *Citrus* species while their relative positions on axis 2 suggest that the eremorange from Corsica has a greater *C. maxima* contribution than the *E. glauca* × *C.* × *sinensis* from Fundecitrus. Taking into account the probable admixture or hybrid status of accessions revealed by the heterozygosity and factorial analysis, the diagnostic SNP mining for the different ancestors was performed using only pure representatives for 10 considered ancestral species. A total of 20,708 diagnostic SNPs were identified and the number of DSNPs for each ancestor is given in the NJ tree of the 14 used accessions (Figure 6). The distribution over the nine chromosomes is available as Supplementary Table 1.

The global contribution of the 10 ancestors to the interspecific hybrids or admixed accessions was estimated from the proportion of specific alleles over the set of DSNPs of each ancestor (Table 1). Contributions below 1.5% were considered as artifactual. The contributions of *C. maxima* and *C. reticulata* to sweet orange, grapefruit, and clementine are in full agreement with previous works (Wu et al., 2014, 2018; Oueslati et al., 2017) as well as the contributions of *C. medica*, *C. maxima*, and *C. reticulata* to lemon (Wu et al., 2018; Ahmed et al., 2019). The two supposed *E. glauca* × *C.* × *sinensis* hybrids displayed a contribution of 50% from *E. glauca*. Both displayed also *C. reticulata* and *C. maxima* contributions with a greater proportion of *C. reticulata* (47%) in the Fundecitrus accession than in the Corsican one (40%). All the finger limes accessions displayed introgressions of *M. australis* in an *M. australasica* background. The estimated contributions of *M. australis* and *M. inodora* to the *M. australis* accession from Fundecitrus are, respectively, 47 and 40%. The *Eremocitrus* × *Microcitrus* accession displayed a major contribution of *M. australasica* (69%) followed by *E. glauca* (23%) and *M. australis* (5.9%). This analysis confirms the absence of *E. glauca* contribution for the supposed *Microcitrus* × *Eremocitrus* hybrid. The *M. papuana* accession displayed a major but incomplete contribution of *M. warburgiana* (65%) without any signal for the other ancestors considered in our study.

**TABLE 1:** Contribution of 10 ancestral species to the admixed and interspecific hybrids of Fundecitrus and Corsican Asian and Oceanian citrus germplasm.

	*C. maxima*	*C. reticulata*	*C. medica.*	*M. garrawayae*	*E. glauca*	*M. australis.*	*M. inodora*	*M. warburgiana*	*M. australasica*	*C. polyandra*
*Citrus* × *sinensis* ‘Tobias’	41.5%	55.9%	0.7%	0.1%	0.1%	0.1%	0.0%	0.1%	0.0%	0.6%
*E. glauca* × *Citrus* sp.	4.3%	47.4%	0.2%	0.1%	50.0%	0.0%	0.1%	0.1%	0.1%	0.6%
*E. glauca* × *Microcitrus* sp.	0.2%	0.5%	0.3%	0.6%	23.2%	5.9%	1.5%	1.7%	68.6%	1.2%
*M. papuana* hybrid	0.0%	0.0%	0.0%	0.0%	0.0%	0.0%	0.0%	64.7%	0.0%	0.0%
*M. australasica* ‘True Sanguinea’	0.2%	0.3%	0.4%	0.5%	0.4%	21.3%	1.8%	0.6%	67.0%	0.9%
*M. australasica* ‘Green’	0.3%	0.3%	0.3%	0.5%	0.3%	23.2%	1.4%	0.4%	65.7%	1.0%
*M. australis* hybrid	0.2%	0.2%	0.7%	0.5%	0.1%	47.2%	39.9%	0.8%	1.4%	0.8%
*Microcitrus* sp. hybrid	0.4%	0.7%	0.3%	0.6%	0.5%	18.1%	1.7%	0.6%	55.6%	1.3%
*Citrus paradisi* ‘Star Ruby’	63.3%	36.4%	0.7%	0.3%	0.0%	0.1%	0.0%	0.1%	0.0%	0.5%
*M. australasica* SRA, Corsica	0.4%	0.3%	0.3%	0.5%	0.3%	17.6%	1.5%	0.5%	66.8%	0.5%
*C.* × *clementina* ‘Nules’	18.1%	77.7%	0.3%	0.1%	0.0%	0.2%	0.1%	0.0%	0.0%	0.4%
Eremorange SRA, Corsica	11.9%	39.8%	0.3%	0.2%	50.1%	0.2%	0.1%	0.1%	0.1%	0.8%
*C.* × *limon* ‘Eureka’	17.9%	33.4%	56.6%	0.1%	0.1%	0.2%	0.0%	0.0%	0.4%	0.9%

**FIGURE 7:**
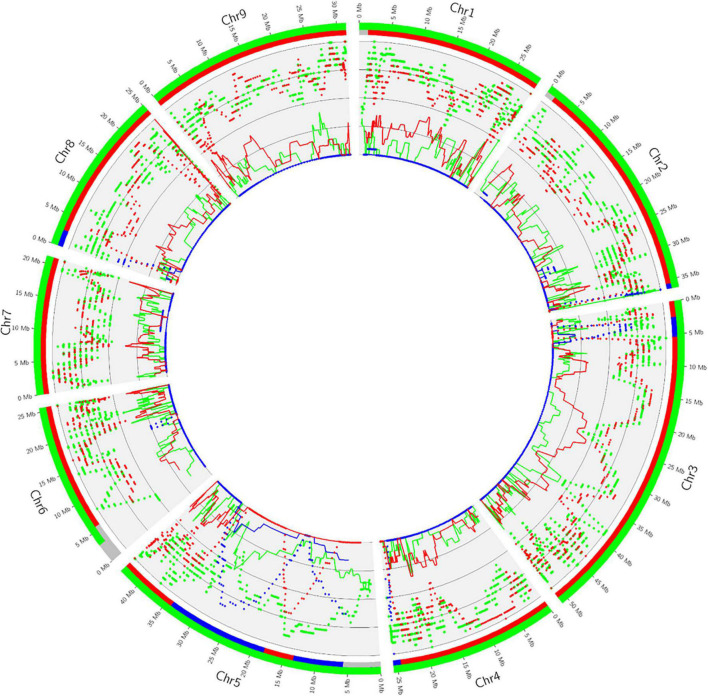
Distribution of heterozygosity (dashes) and homozygosity (line) of the ancestral alleles of *E. glauca* (green), *C. reticulata* (red), and *C. maxima* (blue) along the genome of the supposed *E. glauca* × *C.* × *sinensis* hybrid and phylogenomic karyotype inference (external ribbons with the same color code). Gray corresponds to undetermined regions.

Phylogenomic karyotypes are proposed for the admixed accessions analyzed for HLB resistance. They were inferred from the curve, all along the genome, for homozygosity and heterozygosity for the specific alleles of each ancestor involved in the admixture. Figure 7 provides an example of the supposed *E. glauca* × *C.* × *sinensis* accession. One haplotype is fully constituted by *E. glauca* genome while the other is a mixture of *C. reticulata* and *C. maxima* with a major contribution of *C. reticulata*. This accession is clearly a direct hybrid between *E. glauca* and an Asian variety with C. *reticulata/C. maxima* admixture. The presence of *C. reticulata* heterozygosity signal at the end of chromosome 2, while sweet orange is homozygous for *C. maxima* in the same region, discards sweet orange as the second parent. It is more probable that a cultivated mandarin or a tangor (mandarin × sweet orange hybrid) may be the second parent. The distribution of *C. maxima* genome in the clementine one (Wu et al., 2014) is compatible with the hypothesis that the second parent is a clementine. The Circos representation for *M. australis* hybrid, *Microcitrus* × *Eremocitrus*, *Eremocitrus* × *Microcitrus*, *M. papuana* hybrid, ‘Tobias’ sweet orange and the eremorange from Corsica are given in Supplementary Figure 1. The heterozygosity all along the genome for *M. australis* and *M. inodora* confirms the hypothesis that the *M. australis* accession from Fundecitrus results from a direct hybridization between *M. australis* and *M. inodora*. Among the two accessions supposed to result from admixture between *E. glauca* and *Microcitrus* species, this background is confirmed for the *Eremocitrus* × *Microcitrus* accessions. It displays homozygous regions for *M. australasica* as well as heterozygous areas of *M. australasica*/*M. australis* and *M. australasica/E. glauca*. It may be a hybrid between *M. australasica* and a second parent resulting from a complex admixture between *E. glauca*, *M. australis*, and *M. australasica*. The genome of the supposed *Microcitrus* × *Eremocitrus* hybrid is constituted in large part of *M. australasica* in homozygosity, some regions of *M. australasica/M. australis* heterozygosity, and a small portion of *M. australis* homozygosity at the start of the chromosome 9. *M. papuana* displays a significant signal for *M. warburgiana* heterozygosity in most of the genome but also a signal for *M. warburgiana* homozygosity. If it is a hybrid of *M. papuana*, as suggested by the morphology analysis, this concomitant signal for *M. warburgiana* DSNPs homozygosity and heterozygosity may be explained by the fact that *M. papuana* and *M. warburgiana* share numerous specific mutations differentiating the Papua-New Guinea species from the Australian ones. Indeed, in the absence of a pure representative of *M. papuana* for the DSNPs mining, these Papua-New Guinea-specific SNPs should have been assimilated as *M. warburgiana* DSNPs resulting in a false homozygosity signal for *M. warburgiana* in a hybrid between two Papua-New Guinea species.

**FIGURE 8:**
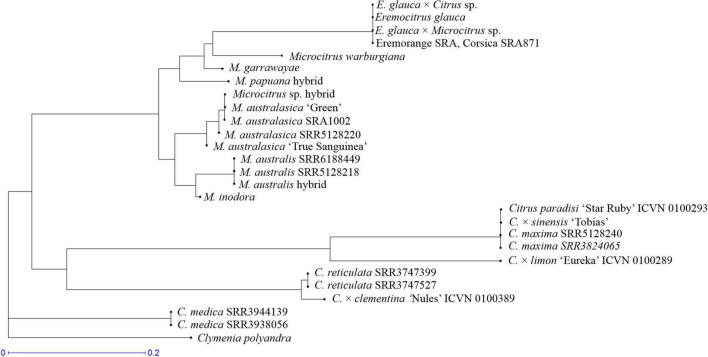
Assignation of the mitochondrial genome of hybrids and admixed Oceanian accessions in relation to ancestral mitochondrial ones; NJ tree based on the genotyping for 40 mitochondrial diallelic DSNPs.

From the NJ tree based on mitochondrial data (Figure 8), it appears that: the *E. glauca* × *C.* × *sinensis* and *Eremocitrus* × *Microcitrus* accessions inherited *E. glauca* mitochondria as well as the eremorange from Corsica. All analyzed finger limes and the *Microcitrus* hybrid accessions have *M. australasica* mitochondria while the *M. australis* from Fundecitrus has *M. australis* mitochondria. The mitochondria of the *M. papuana* accession are differentiated from the *M. warburgiana* one and may be representative of the *M. papuana* species. To summarize, the genotypes used in this study, their common names and phylogenomic constitution after genotyping by sequencing (GBS) analysis are listed in Table 2.

**TABLE 2:** Genotypes used in this study, their common names, and phylogenomic constitution after genotyping by sequencing (GBS) analysis.

Genotype^a^	Common name^b^	After GBS^c^
*Citrus* × *sinensis* (L.) Osbeck ‘Tobias’ (P)^c^	Tobias sweet orange	*C.* × *sinensis* (L.) Osbeck ‘Tobias’
*Microcitrus warburgiana* (F.M. Bailey) Tanaka (M)^d^	New Guinean wild lime	*M. warburgiana* (F.M. Bailey) Tanaka
*M. papuana* Winters (M)	Brown river finger lime	*M. papuana* × *M. warburgiana* hybrid
*M. australis* (A. Cunn. Ex Mudie) Swingle (M)	Australian round lime	*M. australis* × *M. inodora* hybrid
*Eremocitrus glauca* (Lindl.) Swingle (M)	Australian desert lime	*Eremocitrus. glauca*
*Microcitrus* sp. × *E. glauca* hybrid (PM)^e^	Australian lime hybrid BGC 695^b^	*M. australasica* × (*M. australis* × *M. australasica*) hybrid
E. glauca × C. × sinensis (P)	Eremorange	*E. glauca* × *Citrus* sp. hybrid
*E. glauca* × *Microcitrus* sp. (PM)	Australian desert lime hybrid BGC 682^b^	[*E. glauca* × (*M. australis* x *M. australasica*)] × *M. australasica* hybrid

^a^The nomenclature used follows Swingle and Reece (1967) and Bayer et al. (2009) and was also previously used in Alves et al. (2021b). ^b^Original accession number at the Citrus Germplasm Bank (BGC) of Embrapa Cassava and Fruits in Cruz das Almas, Bahia. ^c^P, polyembryonic; ^d^M, monoembryonic; ^e^PM, possibly monoembryonic; According to Swingle and Reece (1967) and Bitters (1986).

### Experiment 1: Las acquisition by *Diaphorina citri* reared on Oceanian citrus genotypes

After 72 h of *D. citri* confinement, none of the new shoot flushes evaluated from plants of all Oceanian citrus genotypes resulted positive by qPCR, while in the case of ‘Tobias’ sweet orange, 100% of the flushes were Las-positive (Table 3). Insects reared on plant flushes followed the same tendency, with none of those fed on Oceanian citrus showing Ct values for Las < 34, but 97.14% (133 of 140) of those fed on ‘Tobias’ sweet orange being clearly Las-positive (*C*t ≤ 34). However, a small number of insects were considered suspicious to be positive as they showed *C*t values between 34 and 36, specifically two of 160 fed on *M. australis* hybrid, seven of 140 on the *Microcitrus* sp. hybrid, six of 220 on the *E. glauca* × *Citrus* sp. hybrid and two of 140 on ‘Tobias’ sweet orange. All insects fed on *M. warburgiana*, *M. papuana* hybrid, *E. glauca*, and *E. glauca* × *Microcitrus* sp. hybrid were Las-negative (Supplementary Table 2).

**TABLE 3:** Acquisition of ‘*Candidatus* Liberibacter asiaticus’ (Las) by *Diaphorina citri* reared for 72 h on new shoot flushes from plants of seven Oceanian citrus genotypes and ‘Tobias’ sweet orange as control, all grafted onto Las-infected ‘Rangpur’ lime rootstocks.

Genotype	Number of plants used for insect acquisition	New shoot flushes		*D. citri* adults	
		Frequency of Las-positive/ total flushes	*C*t avg ± SEM/ Log ± SEM^a^	Frequency of Las-positive/ total insects	*C*t avg ± SEM/ Log ± SEM^b^
*Citrus* × *sinensis* ‘Tobias’	8	(28/28)	27.5 ± 0.55/ 6.1 ± 0.63	(133/145)	31.4 ± 0.14/ 2.3 ± 0.04
*Microcitrus warburgiana*	5	(0/14)	nd^c^	(0/70)	nd
*M. papuana* hybrid	4	(0/16)	nd	(0/80)	nd
*M. australis* hybrid	8	(0/32)	nd	(0/160)	nd
*Eremocitrus glauca*	6	(0/12)	nd	(0/64)	nd
*Microcitrus* sp. hybrid	7	(0/28)	nd	(0/140)	nd
*E. glauca* × *Citrus* sp. hybrid	11	(0/44)	nd	(0/220)	nd
*E. glauca* × *Microcitrus* sp. hybrid	8	(0/32)	nd	(0/160)	nd

^a^Cycle threshold/log10 of amplicon copies per gram of plant tissue average and standard error from all flushes. ^b^Cycle threshold/log10 of amplicon copies per gram of plant tissue average and standard error from all insects. ^c^Non-detected. Plants had been graft-challenge inoculated with Las in both rootstock and scion 30 months earlier. Las titer in log10 of amplicon copies per gram of plant tissue was estimated based on a standard curve as described by Lopes et al. (2013).

### Experiment 2: Las infection in ‘Valencia’ sweet orange plants grafted with bark and budwood from the Oceanian citrus genotypes

Las infection was confirmed in the ‘Rangpur’ lime rootstock bark patches from all composite plants remaining alive with scions of the seven Oceanian citrus genotypes and ‘Tobias’ sweet orange control, with *C*t average values ranging from 28.5 ± 0.91 to 32.6 ± 0.25 (Table 4). Las was also detected in the bark patches of the scions taken at 8 cm above the scion-rootstock union, with Ct average values ranging from 26.4 ± 0.64 to 32.8 ± 0.50, with the exception of all those coming from *E. glauca* which showed *C*t values > 34. Moreover, bark pieces at 8 cm from most *M. warburgiana* and *M. australis* hybrid scions were also Las-negative (Supplementary Table 3). In budwood collected from the apex, Las was detected in all but one (7 of 8) ‘Tobias’ sweet orange, and only two of 11 *E. glauca* × *Citrus* sp. hybrid samples with *C*t average values ranging from 22.5 ± 1.09 to 24.0 ± 0.05, while Las was hardly found in few samples of *M. australis* hybrid (2 of 8), *Microcitrus* sp. hybrid (3 of 7) and *E. glauca* × *Microcitrus* sp. hybrid (1 of 8), with *C*t averages ranging from 30.7 ± 2.74 to 33.6 ± 0.00 in Las-positive samples. Las was not detected in budwood of *M. warburgiana, M. papuana* hybrid, and *E. glauca* scions.

**TABLE 4:** ‘*Candidatus* Liberibacter asiaticus’ (Las) infection, determined by quantitative polymerase chain reaction, in bark patches and budwood collected from different regions (8 cm above the graft union and at the top, respectively) of Oceanian citrus and ‘Tobias’ sweet orange plants, as well as from the stem (5 cm below the graft union) of Las-infected ‘Rangpur’ lime used as rootstock, graft success and Las transmission from those bark patches and budwood to ‘Valencia’ sweet orange plants, 12 and 24 months after grafting.

Genotype		Average *C*t ± SEM^b^ values of Las in bark tissue and budwood grafted onto healthy ‘Valencia’ sweet orange plants			Average *C*t ± SEM values of Las in leaves of the grafted ‘Valencia’ sweet orange plants					
			
	*N* ^a^	Rootstock	Scion		12 months after grafting				24 months after grafting	
					
					Rootstock	Scion		Rootstock	Scion	
			8 cm	Apex		8 cm	Apex		8 cm	Apex
*Citrus* × *sinensis ‘Tobias’*	8	28.8 ± 0.84/ 3.8 ± 0.25 (8/8)	26.4 ± 0.64/ 4.5 ± 0.19 (8/8)	22.5 ± 1.09/ 5.6 ± 0.33 (7/8)	F^c^	21.8 ± 1.19/ 5.9 ± 0.36 (6/8)^d^	29.7 ± 2.13/ 3.5 ± 0.64 (7/8)	F	29.4 ± 0.65/ 3.6 ± 0.20 (6/8)	23.0 ± 1.39/ 5.5 ± 0.42 (7/8)
*Microcitrus warburgiana*	5	29.9 ± 1.1/ 3.4 ± 0.3 (5/5)	30.4 ± 0.00/ 3.3 ± 0.00 (1/5)	nd^b^ (0/5)	F	F	nd (0/5)	F	F	nd (0/5)
*M. papuana* hybrid	4	28.5 ± 0.91/ 3.8 ± 0.27 (4/4)	29.1 ± 1.09/ 3.7 ± 0.33 (4/4)	nd (0/4)	F	F	nd (0/4)	F	F	nd (0/4)
*M. australis* hybrid	8	30.1 ± 1.20/ 3.4 ± 0.36 (8/8)	28.5 ± 0.00/ 3.9 ± 0.00 (1/8)	32.5 ± 0.67/ 2.7 ± 0.20 (2/8)	F	F	nd (0/8)	F	F	nd (0/8)
*Eremocitrus glauca*	7	30.5 ± 1.13/ 3.2 ± 0.34 (7/7)	nd (0/7)	nd (0/7)	F	F	nd (0/7)	F	F	nd (0/7)
*Microcitrus* sp. hybrid	7	29.0 ± 1.37/ 3.7 ± 0.41 (7/7)	27.7 ± 1.00/ 4.1 ± 0.30 (5/7)	30.7 ± 2.74/ 3.2 ± 0.81 (3/7)	F	F	nd (0/7)	F	F	nd (0/7)
*E. glauca* × *Citrus* sp. hybrid	11	28.6 ± 0.85/ 3.8 ± 0.25 (11/11)	29.7 ± 0.50/ 3.5 ± 0.15 (10/11)	24.0 ± 0.05/ 5.2 ± 0.01 (2/11)	F	F	nd (0/11)	F	F	nd (0/11)
*E. glauca* × *Microcitrus* sp. hybrid	8	32.6 ± 0.25/ 2.6 ± 0.08 (8/8)	32.8 ± 0.50/ 2.6 ± 0.15 (2/8)	33.6 ± 0.00/ 2.3 ± 0.00 (1/8)	F	F	nd (0/8)	F	F	nd (0/8)

^a^Number of plants used for the Las graft challenge inoculation experiment (all with Las-positive rootstocks, Ct ≤ 34.0). ^b^Non-detected. ^c^F, grafting failed. ^d^Number of positive ‘Valencia’ sweet orange plants (Ct ≤ 34.0)/total number of indexed ‘Valencia’ sweet orange plants. Las titer in log10 of amplicon copies per gram of plant tissue was estimated based on a standard curve as described by Lopes et al. (2013).

### Experiment 3: Top-grafting of ‘Valencia’ sweet orange plants on scions from the Oceanian citrus genotypes

Budwood from healthy ‘Valencia’ sweet orange plants was grafted on the top of the original plants remaining alive from the Oceanian citrus genotypes and ‘Tobias’ sweet orange growing on Las-infected ‘Rangpur’ lime rootstock. Unfortunately, at the time this experiment was conducted, no plants of *M. warburgiana* were alive, and thus, this genotype was not evaluated. In addition, no ‘Valencia’ sweet orange budwood sprouted on *Microcitrus* sp. hybrid plants (Table 5), likely due to their highly debilitated root system. Rates of budwood sprouting varied from 1/8 for ‘Tobias’ sweet orange, 1/4 for *M. papuana* hybrid, 2/8 for *M. australis* hybrid, 2/6 for *E. glauca*, 4/11 for *E. glauca* × *Citrus* sp. hybrid, and 3/8 for *E. glauca* × *Microcitrus* sp. hybrid. Las was detected only in ‘Valencia’ sweet orange samples top-grafted on the ‘Tobias’ sweet orange with a *C*t of 26.9 (Table 5 and Supplementary Table 4).

**TABLE 5:** Graft success and titer of ‘*Candidatus* Liberibacter asiaticus’ (Las) in new shoots grown from budwood of ‘Valencia’ sweet orange top-grafted on Oceanian citrus genotypes and ‘Tobias’ sweet orange, all growing onto Las-infected ‘Rangpur’ lime rootstocks, as determined through detection of Las16S DNA by qPCR 60 days after bud grafting.

Genotype	*N* ^a^	Ct/Log^b^
*Citrus* × *sinensis* ‘Tobias’	1/8	26.9/4.3
*M. papuana* hybrid	1/4	nd^c^
*M. australis* hybrid	2/8	nd
*Eremocitrus glauca*	2/6	nd
*Microcitrus* sp. hybrid	0/7	
*E. glauca* × *Citrus* sp. hybrid	4/11	nd
*E. glauca* × *Microcitrus* sp. hybrid	3/8	nd

^a^Number of ‘Valencia’ sweet orange indicator sprouts over the total number of bud-grafted plants. ^b^Cycle threshold/log10 of amplicon copies per gram of plant tissue. ^c^Non-detected. Las titer in log10 of amplicon copies per gram of plant tissue was estimated based on a standard curve as described by Lopes et al. (2013).

### Experiment 4: Las multiplication in cuttings of Oceanian citrus genotypes

No plants of *M. warburgiana* or *M. papuana* hybrid were alive to attempt establishing stem cuttings for rooting and all cuttings performed from *M. australis* hybrid, *E. glauca*, and *E. glauca* × *Citrus* sp. hybrid stems did not survive as the root system of the original plants was almost dead by aggressive Las infection of the ‘Rangpur’ lime rootstock. However, 32 and five cutting-derived rooted plants were obtained from *Microcitrus* sp. and *E. glauca* × *Microcitrus* sp. hybrids, respectively, which were all Las-free 10 months after propagation (Table 6 and Supplementary Table 5). As controls, 33 of 39 cutting-derived plants of ‘Tobias’ sweet orange, had *C*t average values between 21.6 ± 0.28 and 29.9 ± 1.13.

**TABLE 6:** ‘*Candidatus* Liberibacter asiaticus’ (Las) infection in leaves of cutting-derived plantlets obtained from Oceanian citrus genotypes and ‘Tobias’ sweet orange control, all grafted onto Las-infected ‘Rangpur’ lime rootstocks, 2 years before and 10 months after cutting propagation.

Genotype	Original plant^a^			Cutting-derived plants	
	*C*t/Log at 24 MAI^b^				
	Plant	Scion leaves	Rootstock	*N* ^c^	Ct avg ± SEM/ Log avg ± SEM^d^
*Citrus* × *sinensis* ‘Tobias’	1	21.2/6.0	30.1/3.4	9/10	24.8 ± 0.34/5.0 ± 0.10
	2	27.9/4.0	29.8/3.5	9/10	29.9 ± 1.13/3.5 ± 0.34
	3	22.9/5.5	30.1/3.4	6/9	28.0 ± 2.87/3.6 ± 0.67
	4	20.3/6.3	29.9/3.4	9/10	21.6 ± 0.28/5.9 ± 0.08
*Microcitrus australis* hybrid	1	nd	30.1/3.4	F^e^	
	2	nd	30.1/3.4	F	
	3	nd	29.9/3.4	F	
	4	nd	30.3/3.3	F	
*Eremocitrus glauca*	1	nd	27.9/4.0	F	
	2	nd	30.3/3.3	F	
	3	nd	31.7/2.9	F	
	4	nd	30.8/3.2	F	
*Microcitrus* sp. hybrid	1	nd	29.0/3.7	0/10	nd^f^
	2	nd	28.9/3.7	0/7	nd
	3	nd	31.7 2.9	0/5	nd
	4	nd	30.1/3.4	0/10	nd
*Eremocitrus glauca* × *Citrus* sp. hybrid	1	nd	29.0/3.7	F	
	2	nd	27.2/4.2	F	
	3	nd	30.6/3.2	F	
	4	nd	29.7/3.5	F	
*E. glauca* × *Microcitrus* sp. hybrid	1	nd	33.4/2.4	0/3	nd
	2	nd	33.0/2.5	0/2	nd
	3	nd	33.3/2.4	F	
	4	nd	33.7/2.3	F	

Las titer in log10 of amplicon copies per gram of plant tissue was estimated based on a standard curve as described by Lopes et al. (2013). ^a^Plants Las-challenged by grafting from which stems were removed to attempt rooting. ^b^Months after graft-challenge Las inoculation. ^c^Number of rooted stem cutting per plant which were evaluated for Las infection. ^d^Cycle threshold average determined through detection of the 16S DNA by qPCR ± Standard error of the mean/log10 of amplicon copies per gram of plant tissue ± standard error of the mean. ^e^Rooting failed. ^*f*^Non-detected.

### Experiment 5: Forcing Las colonization in new shoots of Oceanian citrus genotypes

The stems of all surviving Oceanian citrus plants growing onto Las-infected ‘Rangpur’ lime rootstock were pruned exhaustively in attempts to force Las movement from the rootstock to the new growing tissues in the scions. New shoots grew on stems of most plants, except *E. glauca* × *Microcitrus* sp. hybrid for which only one out of eight plants sprouted. Las was detected in six of the eight ‘Tobias’ plants with an average *C*t value of 26.8 ± 1.48 (Table 7 and Supplementary Table 6), but new shoots from *M. australis* hybrid, *E. glauca, Microcitrus* sp. × *E. glauca* hybrid, and *E. glauca* × *Citrus* sp. hybrid plants were all Las-negative. All original plants of *M. warburgiana* and *M. papuana* hybrid were dead before performing this experiment, as mentioned above.

**TABLE 7:** ‘*Candidatus* Liberibacter asiaticus’ detection in new flushes from Oceanian citrus genotypes and ‘Tobias’ sweet orange as scions onto Las-infected ‘Rangpur’ lime rootstocks, one month after drastic pruning of the canopy at 5 cm above the scion-rootstock union.

Genotypes	*N* ^a^	New flushes after pruning	Freq.^c^
		Ct avg ± SEM^b^	
*Citrus* × *sinensis* ‘Tobias’	6/8	26.8 ± 1.48/4.4 ± 0.45	6/6
*M. australis* hybrid	8/8	nd^d^	0/8
*Eremocitrus glauca*	7/7	nd	0/7
*Microcitrus* sp. hybrid	7/7	nd	0/7
*E. glauca* × *Citrus* sp.	11/11	nd	0/11
*E. glauca* × *Microcitrus* sp. hybrid	1/8	nd	0/1

Las titer in log10 of amplicon copies per gram of plant tissue was estimated based on a standard curve as described by Lopes et al. (2013). ^a^Number of plants that showed new shoot flushes after drastic pruning/total number of original plants that were pruned. ^b^Cycle threshold average determined through detection of the 16S DNA by qPCR/log10 of amplicon copies per gram of plant tissue and standard error of the mean. ^c^Number of qPCR-positive plants (Ct ≤ 34.0)/total number of plants with flushes evaluated. ^d^Non-detected.

## Discussion

In view of the huge losses in citrus production and quality caused by HLB, as well as the high costs of the existing management strategies, finding a durable and sustainable solution for this disease has been the objective of different research programs worldwide. Genetic resistance to HLB has been found in several citrus relatives of the Rutaceae family, Aurantioideae subfamily, when seedling trees in the field were subjected for 6 years to natural disease challenge conditions in Florida, an HLB endemic region (Ramadugu et al., 2016). However, the experimental system did not reveal whether immune germplasm was actually resistance to the bacterium or to the insect vector. For example, the most HLB field-resistant species belonging to the Clauseneae tribe, such as *Clausena lansium* and *Glycosmis pentaphylla*, are resistant to *Diaphorina citri* under controlled greenhouse conditions (Felisberto et al., 2019). Conversely, other Clauseneae species such as *Bergera koenigii* and *Murraya paniculata* are excellent *D. citri* hosts (Tomaseto et al., 2019; Eduardo et al., 2022^3^) but non- or transient Las hosts, respectively (Cifuentes-Arenas et al., 2019; Alves et al., 2021a). Among genera from the true citrus species, partial resistance to *D. citri* has been found in *Poncirus* (Westbrook et al., 2011; Richardson and Hall, 2013; Hall et al., 2015; Felisberto et al., 2019) and in Oceanian *Microcitrus* and *Eremocitrus* species (Eduardo et al., 2022). Therefore, to assess resistance within *Citrus* and *Citrus* relatives to either the bacterium, the insect vector or both, experiments performed under controlled environmental conditions and using challenging procedures rendering unequivocal results are advisable.

In a previous study, after propagation of over 20 citrus relatives within the Citrinae subtribe, Citreae tribe, onto the Las-susceptible ‘Rangpur’ lime rootstock and graft-inoculation with Las-infected budwood on both the scion and rootstock in controlled greenhouse conditions, we selected seven Oceanian citrus genotypes as full-resistant to Las, that is, Las was never detected in their scion leaves over a period of 24 months after inoculation but it did in all rootstocks tested (Alves et al., 2021b). However, as Las was identified through qPCR in the stem tissue at 5 cm above the scion-rootstock union in most plants of the resistant genotypes, and also in stem bark tissue at 30 cm height in a few plants of two of the genotypes under evaluation, we wondered whether these genotypes were actually partial-resistant to Las or transient hosts. Moreover, we performed a phenotypic description and a fine phylogenomic characterization of the seven Oceanian citrus accessions.

The phenotypic analysis of the Fundecitrus accessions considered as representative of *M. papuana* and *M. australis* revealed that they should be rather ascribed as interspecific hybrids. Moreover, the characteristics of the supposed interspecific or intergeneric hybrids did not allow concluding on their origin accurately. Therefore, we performed, by GBS, a fine genetic characterization of their nuclear and mitochondrial genomes in comparison with those of pure representatives of 10 ancestral species of Asian and Oceanian citrus. The identification of 20708 DSNPs allowed us to efficiently infer ancestral karyotypes of the Fundecitrus germplasm, including full-resistant accessions. It extended to Oceanian species the TraceAncestor approach on GBS data proposed by Ahmed et al. (2019) for Asian citrus. The DSNP set should be improved by analyzing several true representatives of the different species and particularly representatives of *M. papuana* that we missed in the present study. In agreement with the phenotypic observations, this analysis confirmed that the *M. inodora, M. garrawayae, M. warburgiana, E. glauca*, and *C. polyandra* accessions of Fundecitrus germplasm were pure representative of these species. According to nuclear and cytoplasmic genome data, the *M. australis* accession should be an *M. australis* × *M. inodora* hybrid while the M. *papuana* accession is a probable admixture between *M. papuana* and *M. warburgiana* with *M. papuana* mitochondria. It would be necessary to include a true *M. papuana* representative in the phylogenomic analysis to conclude this unquestionably. The supposed *E. glauca* × *C.* × *sinensis* appeared to result from an intergeneric cross between *E. glauca* as a female parent and an admixed *C. reticulata/C. maxima* Asian variety as a male parent. However, the phylogenomic profiles discarded sweet orange as a male parent and suggested that the second parent was a clementine. This accession is different from the eremorange from Corsica whose phylogenomic profile is fully compatible with an *E. glauca* × *C.* × *sinensis* origin (Wu et al., 2018). The *Eremocitrus* × *Microcitrus* accession is a complex hybrid involving, in order of importance, *M. australasica, E. glauca*, and *M. australis*. According to the nuclear and mitochondrial data, it should have resulted from [*E. glauca* × (*M. australis* × *M. australasica*)] × *M. australasica* hybridizations. The *Microcitrus* hybrid may have resulted from a kind of backcross *M. australasica* × (*M. australis* × *M. australasica*) with, however, a small *M. australis* introgression in the *M. australasica* recurrent parent. It is interesting to notice that the three Australian finger limes from Fundecitrus and Corsica are not pure *M. australasica* representatives but all of them display *M. australis* introgressions. As a whole, these results highlight the important inclination of the Oceanian species to produce interspecific and even intergeneric hybrids, likely favored by the absence of facultative apomixis in these species. Consequently, this results sometimes in confusing classifications of these accessions in germplasm collections, particularly if the accessions were introduced by seeds. It also stresses the importance of clonal propagation of this germplasm for any analysis of genetic resistance to Las using Oceanian citrus species.

Next, to better characterize the resistance phenotype of the seven full-resistant Oceanian citrus species and hybrids, new experiments with the same plants were designed. We first considered whether *D. citr*i adults were able to acquire Las when fed on Las challenge-inoculated Oceanian citrus scions still surviving on Las-infected ‘Rangpur’ lime rootstocks. Adult *D. citri* can acquire Las within 5–7 h feeding on infected plants (Xu et al., 1988). In our study, after 72 h of confinement on new flushes of the Las-susceptible ‘Tobias’ sweet orange in an environment proper for Las acquisition (Alves et al., 2021a), 97% of the insects acquired the bacteria. Conversely, Las was not detected in any insect fed on the shoot flushes of the seven Oceanian citrus genotypes, which, as expected, were also negative. These results suggested that Las detected in the Oceanian citrus stems did not reach the scions canopy or did at titers insufficient for insect acquisition and then transmission. However, Las was detected in budwood of the canopy of at least some plants from the *M. australis* hybrid and the other three hybrids with *M. australasica* or sweet orange background in concentrations theoretically sufficient to be acquired by *D. citri* and transmitted to other host plants (Lopes and Cifuentes-Arenas, 2021).

Then, we attempted Las transmission by grafting bark pieces and budwood from the Oceanian citrus scions onto ‘Valencia’ sweet orange healthy plants. Although bark grafting failed for all Oceanian species and hybrids, budwood grafting was highly successful in all cases, revealing that Las was efficiently transmitted from Las-positive ‘Tobias’ to ‘Valencia’ sweet orange but, if present, it was not transmitted in any case from the Oceanian citrus genotypes to ‘Valencia’ sweet orange receptor plants. The efficiency of Las transmission by grafting infective budwood is usually high. Notwithstanding, its frequency depends on the bacterial titer in the plant tissue used for grafting, the kind, number, and size of the infected materials used for inoculation, the season of the year, and the environmental conditions (Lopes et al., 2009). The frequency of successful Las transmission in graft-inoculated, greenhouse-grown *C.* × *sinensis* plants of ‘Hamlin’, ‘Natal,’ ‘Pera,’ and ‘Valencia’ sweet orange cultivars was reported to span from 54 to 88% (Lopes et al., 2009; Albrecht et al., 2014). The failure to transmit Las from apical budwood of the Oceanian citrus genotypes suggests that Las detected by qPCR on them was at low titer, with few or no cells alive, and that viable cells were below a minimum threshold number insufficient to be transmitted by grafting.

Afterward, we tried to force Las infection by either top-grafting healthy ‘Valencia’ sweet orange onto the Oceanian citrus scions or by establishing Oceanian citrus scion stem cuttings in pots for rooting. Once either ‘Valencia’ sweet orange or Oceanian citrus cuttings started to grow, they were evaluated for Las detection. However, few plants survived after attempting top-grafting or set up cuttings, mainly because the ‘Rangpur’ lime rootstock was so much compromised by Las infection that Oceanian citrus scions were already dying when these experiments were carried out, about 36–48 months after the challenge inoculation. Even with this, ‘Valencia’ sweet orange plants were Las-negative on *M. papuana* hybrid, *M. australis* hybrid, *E. glauca*, *Microcitrus* sp. hybrid, *E. glauca* × *Citrus* sp. hybrid, and *E. glauca* × *Microcitrus* sp. hybrid remaining alive while only ‘Tobias’ sweet orange interstock was able to infect the top-grafted ‘Valencia’ sweet orange. The graft compatibility of those Las-resistant Oceanian citrus genotypes with a citrus elite variety such as ‘Valencia’ opens the possibility for their use as putatively HLB-resistant rootstocks/interstocks for other citrus cultivars. In citriculture, there are several examples of successful disease control by using resistant/tolerant rootstocks, such as in the cases of citrus tristeza (Garnsey et al., 1987; Yoshida, 1996; Yokomi et al., 2017), citrus sudden death (Román et al., 2004; Yamamoto et al., 2011) and *Phytophthora* spp. gummosis (Wutscher, 1979; Matheron et al., 1998), though for all these scion-rootstock combinations, scion cultivars used should be also disease resistant or tolerant as a premise. For HLB, the situation would be different because there are no citrus cultivars resistant to Las, and the most important varieties are quite sensitive to the bacterium. However, the use of Las-resistant rootstocks may alleviate symptoms and perhaps delay tree decline. Specific experiments should be planned to test this.

On the other hand, rooted cuttings were successfully established only for *Microcitrus* sp. and *E. glauca* × *Microcitrus* sp. hybrids as well as for ‘Tobias’ sweet orange, being 84% of the latter Las-positive while none of the Oceanian citrus hybrid cuttings were Las-infected. The lack of infection in 16% of ‘Tobias’ sweet orange cuttings may be explained by the uneven distribution of Las within the infected cuttings and composite trees (Tatineni et al., 2008; Li et al., 2009; Raiol-Junior et al., 2021a). Lack of Las-infection in rooted cuttings of the Oceanian citrus hybrids remaining alive may be indicative of the inability of the bacterium to accumulate in the phloem of these genotypes and suggested indirectly that erratic detection of Las in Oceanian citrus may result from bacterial movement from the infected rootstock favored under specific physiological conditions.

Aiming to press the canopy of Oceanian citrus to become invaded by viable bacteria from the rootstock, we pruned severely the Oceanian citrus genotypes close to the bud union with the rootstock to promote vigorous scion sprouting and sap movement up with the new flush. Most of the pruned plants produced new growth successfully. Whereas new flushes from ‘Tobias’ sweet orange were all Las-positive with high bacterial titers, those from *M. australis* hybrid, *E. glauca*, and three other Oceanian citrus hybrids were all Las-negative further indicating that the bacterium was unable to infect consistently Oceanian citrus species and hybrids previously considered by us as full-resistant to Las. Under greenhouse conditions, Las moves 2.9–3.9 cm day^–1^ in a citrus host (Raiol-Junior et al., 2021b), preferably toward new flushes and newly developed roots, by following the source:sink driven sap flow (Raiol-Junior et al., 2021a). The presence of Las only in ‘Tobias’ sweet orange shoot flushes after 30 days of sprouting confirmed the viability and the fast movement of Las within the phloem of susceptible plants. On the other hand, the absence of Las in new flushes of *M. australis* hybrid, *E. glauca, Microcitrus* sp. hybrid, *E. glauca* × *Citrus* sp. Hybrid, and *E. glauca* × *Microcitrus* sp. hybrid proved the inability of Las to colonize new tissues of these resistant genotypes.

Overall, these experiments together confirmed that the Oceanian true species, *M. warburgiana* and *E. glauca* together with the *M. papuana* × *M. warburgiana* hybrid were full-resistant to Las, as their canopies resulted Las-negative in all our thorough tests, *D. citri* was unable to acquire Las from their young leaves and attempts to force Las flow from the ‘Rangpur’ lime rootstocks to them as scions or interstocks were unsuccessful. Based on Swingle and Reece (1967) all known species of *Microcitrus* and *Eremocitrus* emerged from a common ancestor likely resembling *M. warburgiana*, the Papua/New Guinea species, from which different lines of evolution produced the quite unique, pronounced xerophytic *E. glauca* and other *Microcitrus* species adapted to different regions of Australia. In the context of resistance to Las, it should be noticed that the two *Microcitrus* species from Papua/New Guinea (*M. warburgiana* and *M. papuana*) as well as *E. glauca* are close phylogenetically according to their mitochondrial genomes and this correlates with these three Oceanian species or hybrids being the most consistently full-resistant to Las, which suggests that full-resistance to Las within Oceanian citrus may have arisen within these few related species.

The response of the other four Oceanian citrus hybrids to Las-aggressive challenge inoculation conceived to compel infection or at least Las translocation from the infected rootstock to the Oceanian hybrids scions was trickier to interpret. Las-positive samples were detected not only close to the rootstock-scion bud unions but also at their canopies, at titers high enough to indicate bacterial infection. As these four genotypes are hybrids between full-resistant and either partial-resistance or susceptible parents, it could be claimed that they may have inherited partial-resistant actually. However, *D. citri* hardly acquired the bacterium from their young leaves and their budwood was unable to transmit Las to ‘Valencia’ sweet orange healthy plants used for biological indexing. This together with the unfeasibility to detect Las in rooted cuttings and in new flushes after hard pruning from some of them suggested that most of the bacterium found in Oceanian citrus hybrid tissues was unviable, likely multiplied at the ‘Rangpur’ lime root systems and moved to sink scion leaves through the phloem vessels under proper physiological conditions and after several years of vascular connections between the heavily Las-infected rootstocks and the Oceanian hybrid scions. Being phloem vessels from Oceanian citrus hybrids incompetent as culture media to support Las survival and multiplication, bacterial cells flowing from the rootstock would progressively decline and die, although unviable bacteria could be detectable by qPCR during months (Josephson et al., 1993). Assessing the cell viability of bacterial pathogens in plant cells is important to understand their interactions and disease progress or resistance responses. There are several methods to study bacterial cell viability, including plating assays, serological techniques, *in vivo* imaging markers, and flow cytometry, among others (Chitarra and van den Bulk, 2003) but they are mostly applicable to culturable bacteria. Although it has been recently proposed a method to discriminate damaged and viable cells from unculturable bacteria in infected plants based on PCR and plant cell dye staining (Sicard et al., 2020), it is not applicable to phloem vessels-restricted Las. Therefore, distinguishing live and dead bacteria is still unresolved for Las. qPCR provides great sensitivity for Las detection but does not allow to get conclusions on bacterial viability. Another characteristic of qPCR detection for Las is that, in our conditions, Ct values higher than 34 produce variable, inconsistent results (Lopes et al., 2013). Moreover, Ct values between 34 and 36 may result from amplification of non-specific genetic material of other microorganisms also living in the phloem (Bao et al., 2019) as high sequence homology between 16S rDNA from Las and citrus-associated bacteria has already been described (Lin et al., 2008). In our study, only Ct values lower than 34 were considered Las-positive to avoid over-interpretation of Las infection in specific samples on plants that resulted Las-negative repeatedly over years.

Consistent and durable full-resistance in the seven Oceanian citrus genotypes together with the proper characterization of their genomic backgrounds and parentages are essential to be able to properly mapping their genomic regions involved in resistance to Las as well as to use them in breeding programs aimed to generate new citrus-like cultivars yielding immunity or high resistance to HLB. The Las-resistance mechanism in Oceanian citrus species and the nature of Las cells detected in budwood from the Oceanian citrus hybrid genotypes are worth further research.

## Data availability statement

The datasets presented in this study can be found in online repositories. The names of the repository/repositories and accession number(s) can be found in the article/Supplementary material.

## Author contributions

MA, LP, EG, NW, SL, and LR-J conceptualized and designed the work. MA, LR-J, and EC collected the data. MM generated GBS data. PO performed genetic and phylogenomic analysis. MA, LR-J, EC, NW, EG, PO, SL, JF, and LP contributed to data analysis and interpretation, drafting, and critical revision of the article. All authors contributed to the article and approved the submitted version.

## Footnotes

1
https://phytozome-next.jgi.doe.gov/info/Cclementina_v1_0

2
https://darwin.cirad.fr/

3 Eduardo, W. I., Silva, A. C., Volpe, H. X. L., Alquézar, B., Peña, L., and Miranda, M. P. (2022). Push-pull and kill strategy for *Diaphorina citri* control in citrus orchards. *Submitted*.
