# Supplementary material for: Insight into resistance to ‘Candidatus Liberibacter asiaticus,’ associated with Huanglongbing, in Oceanian citrus genotypes
Source: Front Plant Sci. 2022 Sep 9;13:1009350. doi: 10.3389/fpls.2022.1009350 (PMC9500433; doi:10.3389/fpls.2022.1009350)
Supplement: Supplementary file 1 [file Data_Sheet_1.docx]

**Supplementary Figure 1** Distribution of heterozygosity (dashes) and homozygosity (line) of the ancestral alleles along the genome of the interspecific hybrids and admixed Oceanian accessions and phylogenomic karyotype inference (external ribbons). *E. glauca*: green; *M. australis*: brown, *M. australasica*: yellow; *M. inodora*: pink; *C. reticulata*: red; *C. maxima*: blue; undetermined: grey.


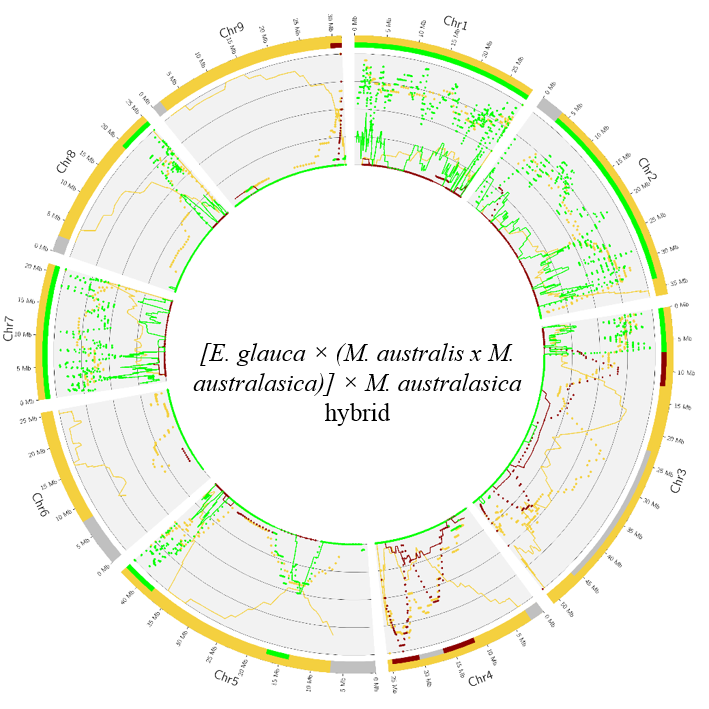

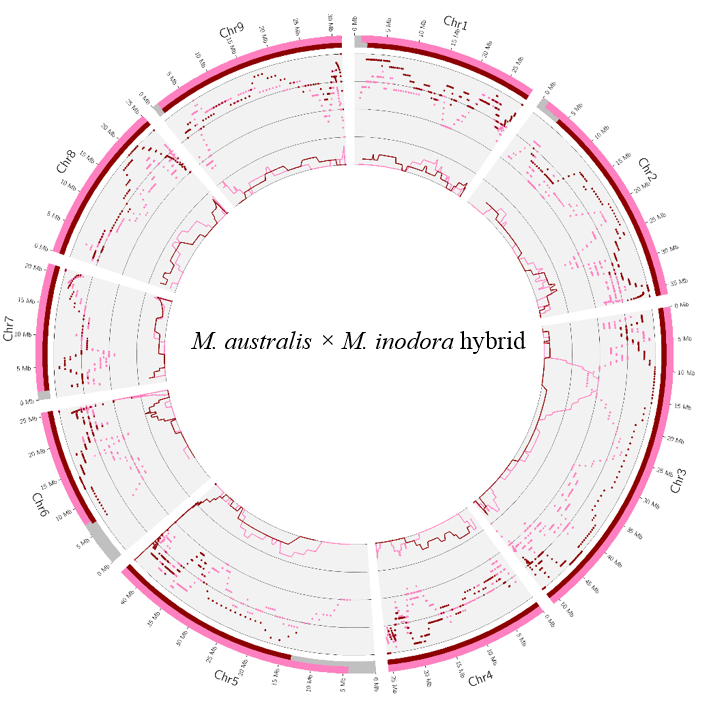


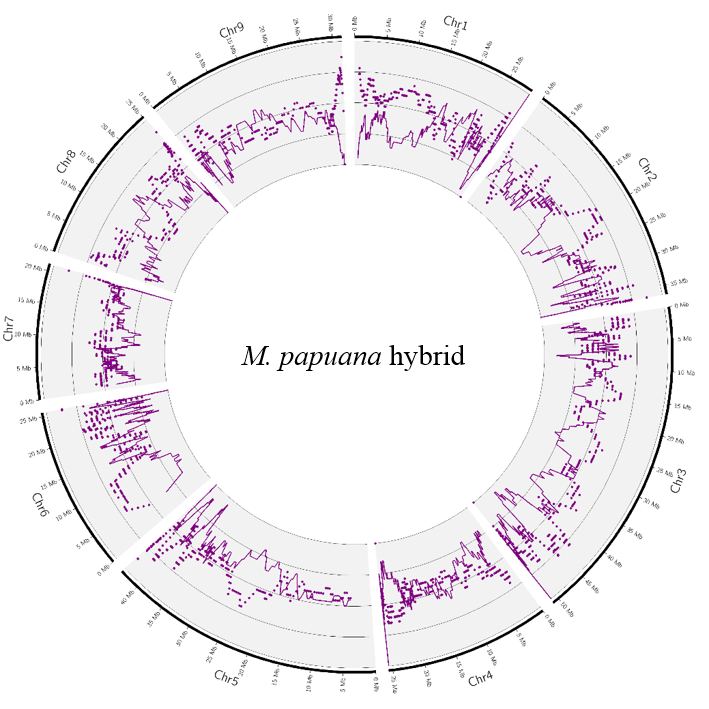


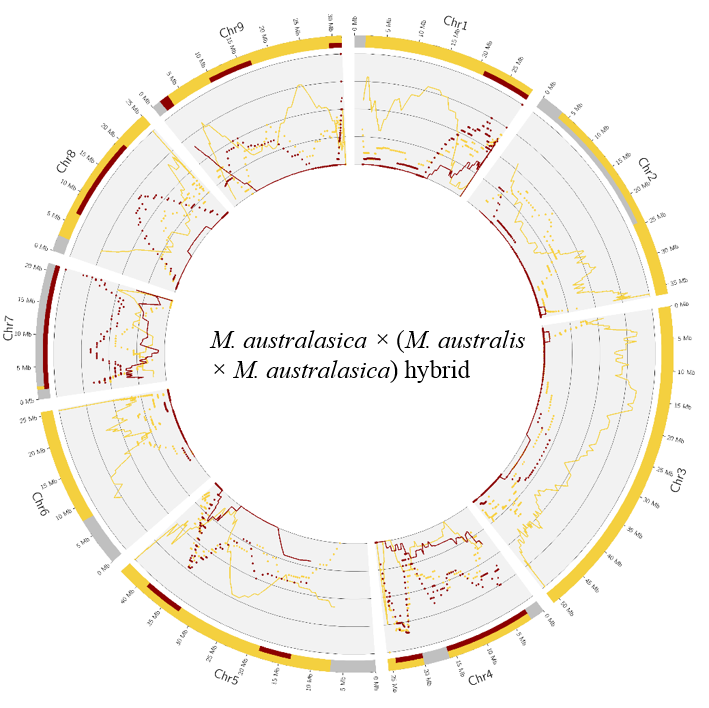


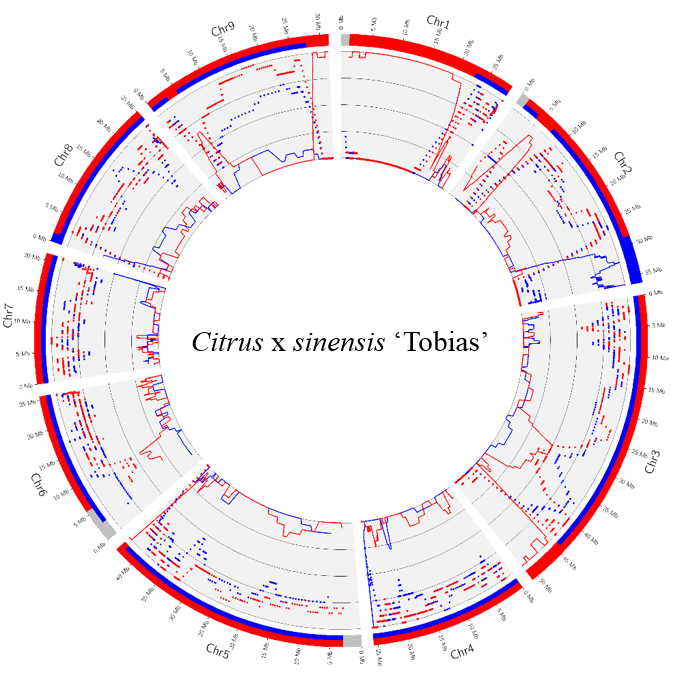

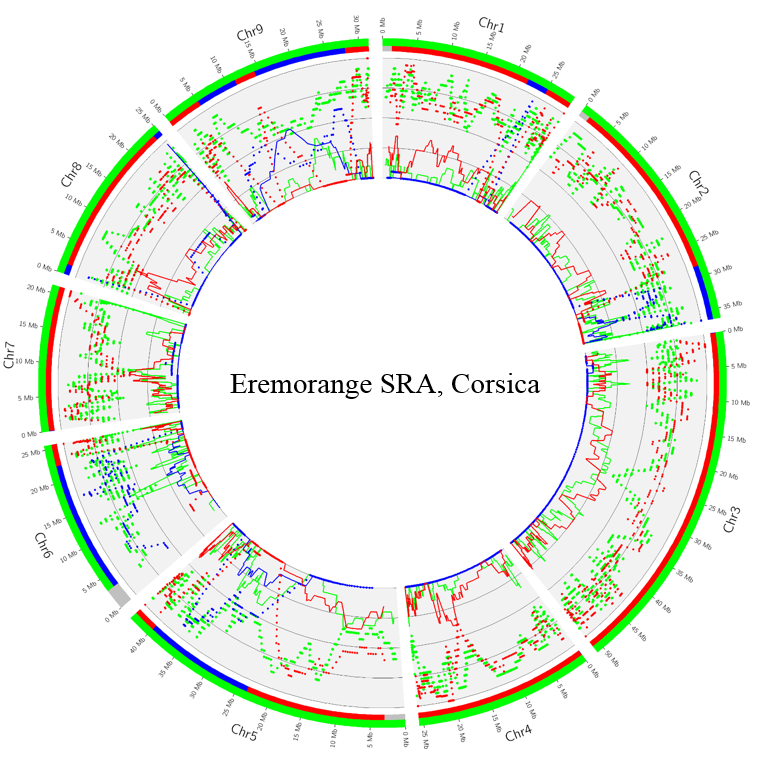


**Supplementary Table 1** Diagnostic SNPs of the Oceanian genotypes and Asian reference species analyzed through genotyping by sequencing

| Genotype/Cromossome | Chr1 | Chr2 | Chr3 | Chr4 | Chr5 | Chr6 | Chr7 | Chr8 | Chr9 | **Total** |
| --- | --- | --- | --- | --- | --- | --- | --- | --- | --- | --- |
| *M. inodora* | 163 | 219 | 282 | 194 | 176 | 149 | 128 | 153 | 150 | **1614** |
| *M. garrawayae* | 272 | 226 | 328 | 229 | 178 | 106 | 94 | 194 | 231 | **1858** |
| *E. glauca* | 588 | 584 | 779 | 460 | 464 | 443 | 437 | 370 | 397 | **4522** |
| *M. australis* | 140 | 123 | 183 | 119 | 113 | 125 | 76 | 84 | 82 | **1045** |
| *C. maxima* | 197 | 253 | 333 | 207 | 199 | 195 | 178 | 120 | 138 | **1820** |
| *M. warburgiana* | 305 | 360 | 493 | 287 | 272 | 303 | 222 | 206 | 240 | **2688** |
| *C. reticulata* | 298 | 341 | 457 | 279 | 245 | 217 | 237 | 235 | 230 | **2539** |
| *C. medica* | 266 | 402 | 567 | 362 | 275 | 204 | 198 | 271 | 315 | **2860** |
| *M. australasica* | 112 | 116 | 198 | 110 | 119 | 97 | 71 | 69 | 89 | **981** |
| *Clymenia polyandra* | 95 | 103 | 120 | 79 | 86 | 70 | 80 | 69 | 79 | **781** |
| **Total** | **2436** | **2727** | **3740** | **2326** | **2127** | **1909** | **1721** | **1771** | **1951** | **20708** |

**Supplementary Table 2** Individual Ct values of acquisition of *'Candidatus* Liberibacter asiaticus' (Las) by *Diaphorina citri* reared for 72 h on new shoot flushes from 4 to 11 plants of seven *C*Las-challenge inoculated Oceanian citrus genotypes and ‘Tobias’ sweet orange as control, all grafted onto Las-infected ‘Rangpur’ lime rootstocks. Plants had been graft-challenge inoculated with Las in both rootstock and scion 30 months earlier.

|  | | **Flush** | | | | |  |  | **Insects** | | | | | | | | | | | | | | | | | | | | |
| --- | --- | --- | --- | --- | --- | --- | --- | --- | --- | --- | --- | --- | --- | --- | --- | --- | --- | --- | --- | --- | --- | --- | --- | --- | --- | --- | --- | --- | --- |
| **Plant^a^** | |  | **Ct^b^** | | | | | | | | | | | | | | | | | | | | | | | | | |  |
|  |  | **1** | | **2** | **3** | **4** |  |  | **1.1** | **1.2** | **1.3** | **1.4** | **1.5** | **2.1** | **2.2** | **2.3** | **2.4** | **2.5** | **3.1** | **3.2** | **3.3** | **3.4** | **3.5** | **4.1** | **4.2** | **4.3** | **4.4** | **4.5** | |
|  | *C. × sinensis* (L.) Osbeck ‘Tobias’ | | | | | | | | | | | | | | | | | | | | | | | | | | | |  |
| 1 | | 27.5 | | 29.7 | 28.2 | ne^c^ |  |  | 29.3 | 30.2 | 32.3 | 31.2 | 33.4 | 28.9 | 31.2 | 30.1 | 33.2 | 31.5 | 27.1 | 30.3 | 29.1 | 32.3 | 33.5 | ne | ne | ne | ne | ne | |
| 2 | | 32.9 | | 33,0 | 31.7 | ne |  |  | 33.2 | 31.2 | 30.1 | 32.3 | 31.2 | 33.5 | 30.2 | 32.1 | 33.1 | 30.2 | 32.1 | 30.1 | 29.1 | 33.1 | 30.2 | ne | ne | ne | ne | ne | |
| 3 | | 25.1 | | 23.4 | 25.4 | 25.7 |  |  | 31.9 | 32.5 | 31.3 | 30.2 | 33.5 | 30.1 | 32.3 | 31.2 | 33.1 | 30.2 | 33.8 | 30.2 | 30.3 | 33.2 | 30.1 | 31.1 | 32.8 | 31 | 33.1 | 30.1 | |
| 4 | | 27,0 | | 25.3 | 22.7 | 30.1 |  |  | 32.1 | 32.3 | 33.2 | 33.4 | 31 | 32.6 | nd | 30.2 | 33.1 | 32 | 35.8 | 30.1 | nd | 30.3 | 33.1 | 30.2 | 33.1 | 29.8 | 28.7 | 30.2 | |
| 5 | | d^d^ | | d | d | d |  |  | d | d | d | d | d | d | d | d | d | d | d | d | d | d | d | d | d | d | d | d | |
| 6 | | 26.0 | | 27.1 | 23.1 | 33.0 |  |  | 33.1 | 31.2 | 33.2 | 30.1 | 32.1 | 30.9 | 31 | nd | 30.1 | 33.2 | 28.7 | 30.7 | 29.8 | 30.2 | 33.4 | 33.1 | 32.2 | 30.8 | 29.8 | 31.8 | |
| 7 | | 26.0 | | 28.0 | 27.0 | ne |  |  | 32.9 | 31.7 | 32.3 | 30.1 | 33.2 | 29.9 | 29 | 28.1 | 30.1 | 30.8 | 30.1 | 27.7 | nd | 30.1 | 32.3 | ne | ne | ne | ne | ne | |
| 8 | | 30.0 | | 28.0 | 27.0 | ne |  |  | 29 | 29.7 | 30.2 | 33.2 | 31.4 | 33.9 | 33.8 | 30.1 | 32 | 33.4 | 32.9 | 28.4 | 32.1 | 33.4 | 30.1 | ne | ne | ne | ne | ne | |
| 9 | | 24.0 | | 29.0 | 26.0 | 29.0 |  |  | 28.2 | 30.2 | 29.5 | 31.9 | 32.7 | 32.9 | 33.2 | 30.1 | 32.3 | 33.8 | 32 | 31.9 | nd | 35.1 | 33.2 | 31.3 | 34 | 30.1 | 30.8 | 32.6 | |
|  | *M. warburgiana* (F.M. Bailey) Tanaka | | | | | | | | | | | | | | | | | | | | | | | | | | | |  |
| 1 | | nd^e^ | | nd | nd | ne |  |  | nd | nd | nd | nd | nd | nd | nd | nd | nd | nd | nd | nd | nd | nd | nd | ne | ne | ne | ne | ne | |
| 2 | | d | | d | d | d |  |  | d | d | d | d | d | d | d | d | d | d | d | d | d | d | d | d | d | d | d | d | |
| 3 | | nd | | nd | nd | ne |  |  | nd | nd | nd | nd | nd | nd | nd | nd | nd | nd | nd | nd | nd | nd | nd | ne | ne | ne | ne | ne | |
| 4 | | d | | d | d | d |  |  | d | d | d | d | d | d | d | d | d | d | d | d | d | d | d | d | d | d | d | d | |
| 5 | | nd | | nd | nd | ne |  |  | nd | nd | nd | nd | nd | nd | nd | nd | nd | nd | nd | nd | nd | nd | nd | ne | ne | ne | ne | ne | |
| 6 | | d | | d | d | d |  |  | d | d | d | d | d | d | d | d | d | d | d | d | d | d | d | d | d | d | d | d | |
| 7 | | nd | | nd | ne | ne |  |  | nd | nd | nd | nd | nd | nd | nd | nd | nd | nd | ne | ne | ne | ne | ne | ne | ne | ne | ne | ne | |
| 8 | | nd | | nd | nd | ne |  |  | nd | nd | nd | nd | nd | nd | nd | nd | nd | nd | nd | nd | nd | nd | nd | ne | ne | ne | ne | ne | |
| 9 | | d | | d | d | d |  |  | d | d | d | d | d | d | d | d | d | d | d | d | d | d | d | d | d | d | d | d | |
|  | *M. papuana* hybrid | | | | | | | | | | | | | | | | | | | | | | | | | | | |  |
| 1 | | d | | d | d | d |  |  | d | d | d | d | d | d | d | d | d | d | d | d | d | d | d | d | d | d | d | d | |
| 2 | | nd | | nd | nd | nd |  |  | nd | nd | nd | nd | nd | nd | nd | nd | nd | nd | nd | nd | nd | nd | nd | nd | nd | nd | nd | nd | |
| 3 | | nd | | nd | nd | nd |  |  | nd | nd | nd | nd | nd | nd | nd | nd | nd | nd | nd | nd | nd | nd | nd | nd | nd | nd | nd | nd | |
| 4 | | nd | | nd | nd | nd |  |  | nd | nd | nd | nd | nd | nd | nd | nd | nd | nd | nd | nd | nd | nd | nd | nd | nd | nd | nd | nd | |
| 5 | | d | | d | d | d |  |  | d | d | d | d | d | d | d | d | d | d | d | d | d | d | d | d | d | d | d | d | |
| 6 | | d | | d | d | d |  |  | d | d | d | d | d | d | d | d | d | d | d | d | d | d | d | d | d | d | d | d | |
| 7 | | nd | | nd | nd | nd |  |  | nd | nd | nd | nd | nd | nd | nd | nd | nd | nd | nd | nd | nd | nd | nd | nd | nd | nd | nd | nd | |
| 8 | | d | | d | d | d |  |  | d | d | d | d | d | d | d | d | d | d | d | d | d | d | d | d | d | d | d | d | |
|  | *M. australis* hybrid | | | | | | | | | | | | | | | | | | | | | | | | | | | |  |
| 1 | | d | | d | d | d |  |  | d | d | d | d | d | d | d | d | d | d | d | d | d | d | d | d | d | d | d | d | |
| 2 | | nd | | nd | nd | nd |  |  | nd | nd | nd | nd | nd | nd | nd | nd | nd | nd | nd | nd | nd | nd | nd | nd | nd | nd | nd | nd | |
| 3 | | nd | | nd | nd | nd |  |  | nd | nd | nd | nd | nd | nd | nd | 35.7 | nd | nd | nd | nd | nd | nd | nd | nd | nd | nd | nd | nd | |
| 4 | | nd | | nd | nd | nd |  |  | nd | nd | nd | nd | nd | nd | nd | nd | nd | nd | nd | nd | nd | nd | nd | nd | nd | nd | nd | nd | |
| 5 | | nd | | nd | nd | nd |  |  | nd | nd | nd | nd | nd | nd | nd | nd | nd | nd | nd | nd | nd | nd | nd | nd | nd | nd | nd | nd | |
| 6 | | nd | | nd | nd | nd |  |  | nd | nd | nd | nd | nd | nd | nd | nd | nd | nd | nd | nd | 35.1 | nd | nd | nd | nd | nd | nd | nd | |
| 7 | | nd | | nd | nd | nd |  |  | nd | nd | nd | nd | nd | nd | nd | nd | nd | nd | nd | nd | nd | nd | nd | nd | nd | nd | nd | nd | |
| 8 | | d | | d | d | d |  |  | d | d | d | d | d | d | d | d | d | d | d | d | d | d | d | d | d | d | d | d | |
| 9 | | nd | | nd | nd | nd |  |  | nd | nd | nd | nd | nd | nd | nd | nd | nd | nd | nd | nd | nd | nd | nd | nd | nd | nd | nd | nd | |
| 10 | | nd | | nd | nd | nd |  |  | nd | nd | nd | nd | nd | nd | nd | nd | nd | nd | nd | nd | nd | nd | nd | nd | nd | nd | nd | nd | |
|  | *E. glauca* (Lindl.) Swingle | | | | | | | | | | | | | | | | | | | | | | | | | | | |  |
| 1 | | nd | | nd | nd | ne |  |  | nd | nd | nd | nd | nd | nd | nd | nd | nd | nd | nd | nd | nd | nd | nd | ne | ne | ne | ne | ne | |
| 2 | | nd | | nd | nd | ne |  |  | nd | nd | nd | nd | nd | nd | nd | nd | nd | nd | nd | nd | nd | nd | nd | ne | ne | ne | ne | ne | |
| 3 | | nd | | nd | nd | ne |  |  | nd | nd | nd | nd | nd | nd | nd | nd | nd | nd | nd | nd | nd | nd | nd | ne | ne | ne | ne | ne | |
| 4 | | nd | | nd | nd | ne |  |  | nd | nd | nd | nd | nd | nd | nd | nd | nd | nd | nd | nd | nd | nd | nd | ne | ne | ne | ne | ne | |
| 5 | | nd | | nd | nd | ne |  |  | nd | nd | nd | nd | nd | nd | nd | nd | nd | nd | nd | nd | nd | nd | nd | ne | ne | ne | ne | ne | |
| 6 | | ne | | ne | ne | ne |  |  | ne | ne | ne | ne | ne | ne | ne | ne | ne | ne | ne | ne | ne | ne | ne | ne | ne | ne | ne | ne | |
| 7 | | nd | | nd | nd | ne |  |  | nd | nd | nd | nd | nd | nd | nd | nd | nd | nd | nd | nd | nd | nd | nd | ne | ne | ne | ne | ne | |
|  | *Microcitrus* sp. hybrid | | | | | | | | | | | | | | | | | | | | | | | | | | | |  |
| 1 | | nd | | nd | nd | nd |  |  | nd | nd | nd | nd | nd | nd | nd | nd | nd | nd | nd | nd | nd | nd | nd | nd | nd | nd | nd | nd | |
| 2 | | nd | | nd | nd | nd |  |  | nd | nd | nd | nd | nd | nd | nd | nd | nd | nd | nd | nd | nd | nd | nd | nd | nd | 34.9 | nd | nd | |
| 3 | | nd | | nd | nd | nd |  |  | nd | nd | nd | nd | nd | nd | nd | nd | nd | nd | nd | nd | nd | nd | nd | nd | nd | 34.8 | nd | nd | |
| 4 | | nd | | nd | nd | nd |  |  | nd | nd | nd | nd | nd | nd | nd | nd | nd | nd | nd | nd | nd | nd | nd | nd | nd | nd | nd | nd | |
| 5 | | nd | | nd | nd | nd |  |  | nd | nd | 35.5 | nd | nd | nd | nd | nd | nd | nd | nd | nd | 35.3 | nd | nd | nd | nd | nd | nd | nd | |
| 6 | | nd | | nd | nd | nd |  |  | nd | nd | nd | nd | nd | nd | nd | nd | nd | nd | nd | nd | nd | nd | nd | nd | nd | nd | nd | nd | |
| 7 | | nd | | nd | nd | nd |  |  | nd | nd | 35.9 | nd | nd | nd | nd | nd | nd | nd | nd | nd | 34.2 | 34.5 | nd | nd | nd | nd | nd | nd | |
|  | *E. glauca* x *Citrus* sp. hybrid | | | | | | | | | | | | | | | | | | | | | | | | | | | |  |
| 1 | | nd | | nd | nd | nd |  |  | nd | nd | nd | nd | nd | nd | nd | nd | nd | nd | nd | nd | nd | nd | nd | nd | nd | nd | nd | nd | |
| 2 | | nd | | nd | nd | nd |  |  | nd | nd | nd | nd | nd | nd | nd | nd | nd | nd | nd | nd | nd | nd | nd | nd | nd | nd | nd | 34.8 | |
| 3 | | nd | | nd | nd | nd |  |  | nd | nd | nd | nd | nd | nd | nd | nd | nd | nd | nd | nd | nd | nd | nd | nd | nd | nd | nd | nd | |
| 4 | | nd | | nd | nd | nd |  |  | nd | nd | nd | nd | nd | nd | nd | nd | nd | nd | nd | nd | nd | nd | nd | nd | nd | nd | nd | nd | |
| 5 | | nd | | nd | nd | nd |  |  | nd | nd | nd | nd | nd | 35.6 | nd | nd | nd | nd | nd | nd | nd | nd | nd | nd | nd | nd | nd | nd | |
| 6 | | nd | | nd | nd | nd |  |  | nd | nd | nd | nd | nd | nd | nd | nd | nd | nd | nd | nd | nd | nd | nd | nd | nd | nd | nd | nd | |
| 7 | | nd | | nd | nd | nd |  |  | nd | nd | nd | nd | nd | nd | nd | nd | nd | nd | nd | nd | nd | nd | nd | nd | nd | nd | nd | nd | |
| 8 | | d | | d | d | d |  |  | d | d | d | d | d | d | d | d | d | d | d | d | d | d | d | d | d | d | d | d | |
| 9 | | nd | | nd | nd | nd |  |  | nd | nd | nd | nd | nd | nd | nd | nd | nd | nd | nd | nd | nd | nd | nd | nd | nd | nd | nd | nd | |
| 10 | | nd | | nd | nd | nd |  |  | nd | nd | nd | nd | nd | nd | nd | nd | nd | nd | nd | nd | nd | nd | nd | nd | nd | nd | nd | nd | |
| 11 | | nd | | nd | nd | nd |  |  | nd | 35.1 | nd | nd | nd | nd | nd | nd | nd | nd | nd | nd | nd | nd | nd | 35.8 | nd | nd | nd | nd | |
| 12 | | nd | | nd | nd | nd |  |  | nd | 35.9 | nd | nd | nd | nd | nd | nd | nd | nd | nd | nd | nd | nd | nd | nd | 36.0 | nd | nd | nd | |
|  | *E. glauca* x *Microcitrus* sp. hybrid | | | | | | | | | | | | | | | | | | | | | | | | | | | |  |
| 1 | | nd | | nd | nd | nd |  |  | nd | nd | nd | nd | nd | nd | nd | nd | nd | nd | nd | nd | nd | nd | nd | nd | nd | nd | nd | nd | |
| 2 | | nd | | nd | nd | nd |  |  | nd | nd | nd | nd | nd | nd | nd | nd | nd | nd | nd | nd | nd | nd | nd | nd | nd | nd | nd | nd | |
| 3 | | nd | | nd | nd | nd |  |  | nd | nd | nd | nd | nd | nd | nd | nd | nd | nd | nd | nd | nd | nd | nd | nd | nd | nd | nd | nd | |
| 4 | | nd | | nd | nd | nd |  |  | nd | nd | nd | nd | nd | nd | nd | nd | nd | nd | nd | nd | nd | nd | nd | nd | nd | nd | nd | nd | |
| 5 | | nd | | nd | nd | nd |  |  | nd | nd | nd | nd | nd | nd | nd | nd | nd | nd | nd | nd | nd | nd | nd | nd | nd | nd | nd | nd | |
| 6 | | nd | | nd | nd | nd |  |  | nd | nd | nd | nd | nd | nd | nd | nd | nd | nd | nd | nd | nd | nd | nd | nd | nd | nd | nd | nd | |
| 7 | | nd | | nd | nd | nd |  |  | nd | nd | nd | nd | nd | nd | nd | nd | nd | nd | nd | nd | nd | nd | nd | nd | nd | nd | nd | nd | |
| 8 | | nd | | nd | nd | nd |  |  | nd | nd | nd | nd | nd | nd | nd | nd | nd | nd | nd | nd | nd | nd | nd | nd | nd | nd | nd | nd | |

^a^Plant propagation evaluated (with Las-positive rootstocks, Ct ≤ 34.0).

^b^Cycle threshold determined through detection of the 16S DNA from Las by qPCR.

^c^Not-evaluated.

^d^Plant died before starting this experiment and .

^e^Non-detected.

**Supplementary Table 3** Values per plant of quantitative polymerase chain reaction results of bark patches collected from different regions of *'Candidatus* Liberibacter asiaticus' (Las)-resistant Oceanian citrus genotypes and HLB-susceptible ‘Tobias’ sweet orange, all growing onto Las-infected ‘Rangpur’ lime rootstocks, and transmission frequencies of Las from the bark patches to ‘Valencia’ sweet orange plants, 12 and 24 months after inoculation by grafting.

|  | **Tissue grafted** | | | **Sweet Orange Leaves** | | | | | |
| --- | --- | --- | --- | --- | --- | --- | --- | --- | --- |
| **Plant** | **Rootstock** | **Scion** | | **12 MAI^a^** | | | **24 MAI** | | |
|  |  |  |  | **Rootstock** | **Scion** | | **Rootstock** | **Scion** | |
|  |  | **8 cm** | **Apex** |  | **8 cm** | **Apex** |  | **8 cm** | **Apex** |
| *C. × sinensis* (L.) Osbeck ‘Tobias’ | | | | | | | | | |
| 1 | 26.8 | 25.1 | 19.9 | F^b^ | F | 23.1 | F | F | 21.7 |
| 2 | 28.4 | 24.6 | 22.9 | F | nd^d^ | 32.3 | F | nd | 31.2 |
| 3 | 26.1 | 25.1 | 21.5 | F | nd | 20.1 | F | nd | 21.5 |
| 4 | 30.2 | 27.2 | 35.6 | F | F | nd | F | nd | nd |
| 5 | d^c^ | d | d | d | d | d | d | d | d |
| 6 | 29.4 | 26.2 | 24.6 | F | 21.0 | 33.0 | F | 29.3 | 21.1 |
| 7 | 30.3 | 27.6 | 24.6 | F | 22.3 | 33.5 | F | 29.4 | 22.5 |
| 8 | 32.9 | 30.0 | 26.0 | F | 24.7 | 33.2 | F | 27.9 | 22.1 |
| 9 | 26.3 | 25.2 | 18.0 | F | 19.0 | 33.0 | F | 31.1 | 20.8 |
| *M. warburgiana* (F.M. Bailey) Tanaka | | | | | | | | | |
| 1 | 27.4 | 30.4 | nd | F | F | nd | F | F | nd |
| 2 | d | d | d | d | d | d | d | d | d |
| 3 | 27.5 | nd | nd | F | F | nd | F | F | nd |
| 4 | d | d | d | d | d | d | d | d | d |
| 5 | 30.1 | nd | nd | F | F | nd | F | F | nd |
| 6 | d | d | d | d | d | d | d | d | d |
| 7 | 31.2 | nd | nd | F | F | nd | F | F | nd |
| 8 | 33.2 | nd | nd | F | F | nd | F | F | nd |
| 9 | d | d | d | d | d | d | d | d | d |
| *M. papuana* hybrid | | | | | | | | | |
| 1 | d | d | d | d | d | d | d | d | d |
| 2 | 27.1 | 30.3 | 35.0 | F | F | nd | F | F | nd |
| 3 | 29.2 | 28.7 | nd | F | F | nd | F | F | nd |
| 4 | 30.8 | 31.2 | 35.6 | F | F | nd | F | F | nd |
| 5 | d | d | d | d | d | d | d | d | d |
| 6 | d | d | d | d | d | d | d | d | d |
| 7 | 27.0 | 26.2 | 35.7 | F | F | nd | F | - | nd |
| 8 | d | d | d | d | d | d | d | d | d |
| *M. australis* hybrid | | | | | | | | | |
| 1 | d | d | d | d | d | d | d | d | d |
| 2 | 25.3 | nd | nd | F | F | nd | F | F | nd |
| 3 | 29.4 | 28.5 | 35.6 | F | F | nd | F | F | nd |
| 4 | 29.4 | nd | nd | F | F | nd | F | F | nd |
| 5 | 33.9 | 35.0 | 33.1 | F | F | nd | F | F | nd |
| 6 | 33.2 | nd | nd | F | F | nd | F | F | nd |
| 7 | 25.8 | nd | nd | F | F | nd | F | F | nd |
| 8 | d | d | d | d | d | d | d | d | d |
| 9 | 30.0 | 35.7 | 31.8 | F | F | nd | F | F | nd |
| 10 | 33.8 | nd | nd | F | F | nd | F | F | nd |
| *E. glauca* (Lindl.) Swingle | | | | | | | | | |
| 1 | 31.8 | nd | nd | F | F | nd | F | F | nd |
| 2 | 27.4 | nd | nd | F | F | nd | F | F | nd |
| 3 | 27.1 | nd | nd | F | F | nd | F | F | nd |
| 4 | 27.6 | 35.3 | nd | F | F | nd | F | F | nd |
| 5 | 33.1 | 35.8 | nd | F | F | nd | F | F | nd |
| 6 | 32.9 | nd | nd | F | F | nd | F | F | nd |
| 7 | 33.7 | nd | nd | F | F | nd | F | F | nd |
| *Microcitrus* sp. hybrid | | | | | | | | | |
| 1 | 25.6 | 26.9 | 24.4 | F | F | nd | F | F | nd |
| 2 | 25.2 | 25.7 | nd | F | F | nd | F | F | nd |
| 3 | 33.2 | 35.2 | 36.0 | F | F | nd | F | F | nd |
| 4 | 29.5 | 27.7 | 33.8 | F | F | nd | F | F | nd |
| 5 | 29.9 | 31.4 | 35.0 | F | F | nd | F | F | nd |
| 6 | 34.0 | nd | nd | F | F | nd | F | F | nd |
| 7 | 26.0 | 26.6 | 33.9 | F | F | nd | F | F | nd |
| *E. glauca* x *Citrus* sp. hybrid | | | | | | | | | |
| 1 | 26.0 | 28.8 | nd | F | F | nd | F | F | nd |
| 2 | 30.7 | 28.6 | 23.9 | F | F | nd | F | F | nd |
| 3 | 33.5 | 30.4 | nd | F | F | nd | F | F | nd |
| 4 | 26.6 | 30.3 | nd | F | F | nd | F | F | nd |
| 5 | 25.9 | 30.2 | nd | F | F | nd | F | F | nd |
| 6 | 33.6 | nd | nd | F | F | nd | F | F | nd |
| 7 | 28.3 | 28.7 | 35.5 | F | F | nd | F | F | nd |
| 8 | d | d | d | d | d | d | d | d | d |
| 9 | 28.5 | 27.4 | nd | F | F | nd | F | F | nd |
| 10 | 26.7 | 28.2 | nd | F | F | nd | F | F | nd |
| 11 | 28.1 | 32.2 | 24.0 | F | F | nd | F | F | nd |
| 12 | 26.7 | 31.8 | 35.0 | F | F | nd | F | F | nd |
| *E. glauca* x *Microcitrus* sp. hybrid | | | | | | | | | |
| 1 | 32.1 | 34.4 | 33.6 | F | F | nd | F | F | nd |
| 2 | 32.9 | 35.4 | nd | F | F | nd | F | F | nd |
| 3 | 31.9 | 35.5 | nd | F | F | nd | F | F | nd |
| 4 | 32.2 | nd | nd | F | F | nd | F | F | nd |
| 5 | 32.0 | 35.7 | nd | F | F | nd | F | F | nd |
| 6 | 33.1 | 32.3 | nd | F | F | nd | F | F | nd |
| 7 | 32.7 | 33.3 | nd | F | F | nd | F | F | nd |
| 8 | 34.0 | nd | nd | F | F | nd | F | F | nd |

^a^ Months after inoculation

^b^ Grafting for indexing failed.

^c^ Plant died and evaluation was not possible to be done.

^d^ Non-detected.

**Supplementary Table 4** Graft success and Ct values of *'Candidatus* Liberibacter asiaticus' (Las) to new shoots grown from budwood of ‘Valencia’ sweet orange top-grafted on Oceanian citrus genotypes and ‘Tobias’ sweet orange, all growing onto Las-infected ‘Rangpur’ lime rootstocks, as determined through detection of *C*Las16S DNA by qPCR 60 days after bud grafting.

| **Plant**^a^ | **‘Valencia’ sweet orange leaves top-grafted** |
| --- | --- |
| *C. × sinensis* (L.) Osbeck ‘Tobias’ | |
| 1 | F^b^ |
| 2 | F |
| 3 | F |
| 4 | F |
| 5 | d^c^ |
| 6 | 26.9 |
| 7 | F |
| 8 | F |
| 9 | F |
| *M. warburgiana* (F.M. Bailey) Tanaka | |
| 1 | F |
| 2 | d |
| 3 | F |
| 4 | d |
| 5 | F |
| 6 | d |
| 7 | F |
| 8 | F |
| 9 | d |
| *M. papuana* hybrid | |
| 1 | d |
| 2 | F |
| 3 | nd^d^ |
| 4 | F |
| 5 | d |
| 6 | d |
| 7 | F |
| 8 | d |
| *M. australis* hybrid | |
| 1 | d |
| 2 | F |
| 3 | F |
| 4 | nd |
| 5 | F |
| 6 | F |
| 7 | nd |
| 8 | d |
| 9 | F |
| 10 | F |
| *E. glauca* (Lindl.) Swingle | |
| 1 | F |
| 2 | F |
| 3 | nd |
| 4 | F |
| 5 | F |
| 6 | F |
| 7 | nd |
| *Microcitrus* sp. hybrid | |
| 1 | F |
| 2 | F |
| 3 | F |
| 4 | F |
| 5 | F |
| 6 | F |
| 7 | F |
| *E. glauca* x *Citrus* sp. hybrid | |
| 1 | nd |
| 2 | nd |
| 3 | F |
| 4 | F |
| 5 | F |
| 6 | F |
| 7 | nd |
| 8 | d |
| 9 | F |
| 10 | nd |
| 11 | F |
| 12 | F |
| *E. glauca* x *Microcitrus* sp. hybrid | |
| 1 | F |
| 2 | nd |
| 3 | F |
| 4 | F |
| 5 | F |
| 6 | F |
| 7 | nd |
| 8 | nd |

^a^ Plant evaluated (with Las-positive rootstocks, Ct ≤ 34.0).

^b^ Top-grafting failed.

^c^ Plant died and evaluation was not possible to be done.

^d^ Non-detected.

**Supplementary Table 5** *'Candidatus* Liberibacter asiaticus' (Las) Ct values in scion leaves and rootstock 24 months after graft-inoculation and in leaves of cutting-derived plantlets obtained from Oceanian citrus genotypes and ‘Tobias’ sweet orange control, all grafted onto Las-infected ‘Rangpur’ lime rootstocks, ten months after cutting propagation.

| **Genotype** | **Original Plant^a^** | | |  | **Cutting-derived plants** | | | | | | | | | |
| --- | --- | --- | --- | --- | --- | --- | --- | --- | --- | --- | --- | --- | --- | --- |
|  | **Ct at 24 MAI^b^** | | |  |  |  |  |  |  |  |  |  |  |  |
|  | **Plant** | **Scion leaves** | **Rootstock** |  | **Ct of cuttings repetitions** | | | | | | | | | |
|  |  |  |  |  | **1** | **2** | **3** | **4** | **5** | **6** | **7** | **8** | **9** | **10^c^** |
| *Citrus* × *sinensis ‘*Tobias’ | 1 | 21.2 | 30.1 |  | 23.5 | 25.9 | 25.3 | 24.9 | 23.1 | 24.1 | 25.5 | 24.8 | nd | 26.1 |
|  | 2 | 27.9 | 29.8 |  | 27.3 | 30.3 | 25.8 | nd^e^ | 29.1 | 27.4 | 28.5 | 30.7 | 30.4 | 29.2 |
|  | 3 | 22.9 | 30.1 |  | 26.4 | 25.1 | nd | nd | nd | nd | 35.0 | nd | nd | 30.4 |
|  | 4 | 20.3 | 29.9 |  | 21.7 | 22.5 | 20.0 | 21.3 | 20.7 | 22.0 | 21.3 | 22.3 | 23.0 | 21.4 |
| *Microcitrus warburgiana* | *** |  | | | | |  |  |  |  |  |  |  |  |
| *M. papuana* hybrid | *** |  | | | | |  |  |  |  |  |  |  |  |
| *M. australis* hybrid | 1 | nd | 30.1 |  | F^e^ | |  |  |  |  |  |  |  |  |
|  | 2 | nd | 30.1 |  | F | |  |  |  |  |  |  |  |  |
|  | 3 | nd | 29.9 |  | F | |  |  |  |  |  |  |  |  |
|  | 4 | nd | 30.3 |  | F | |  |  |  |  |  |  |  |  |
| *Eremocitrus glauca* | 1 | nd | 27.9 |  | F | |  |  |  |  |  |  |  |  |
|  | 2 | nd | 30.3 |  | F | |  |  |  |  |  |  |  |  |
|  | 3 | nd | 31.7 |  | F | |  |  |  |  |  |  |  |  |
|  | 4 | nd | 30.8 |  | F | |  |  |  |  |  |  |  |  |
| *Microcitrus* sp. hybrid | 1 | nd | 29.0 |  | nd | nd | nd | nd | nd | nd | nd | nd | nd | nd |
|  | 2 | nd | 28.9 |  | nd | nd | nd | nd | nd | nd | nd |  |  |  |
|  | 3 | nd | 31.7 |  | nd | nd | nd | nd | nd |  |  |  |  |  |
|  | 4 | nd | 30.1 |  | nd | nd | nd | nd | nd | nd | nd | nd | nd | nd |
| *Eremocitrus glauca × Citrus* sp. hybrid | 1 | nd | 29.0 |  | F | |  |  |  |  |  |  |  |  |
|  | 2 | nd | 27.2 |  | F | |  |  |  |  |  |  |  |  |
|  | 3 | nd | 30.6 |  | F | |  |  |  |  |  |  |  |  |
|  | 4 | nd | 29.7 |  | F | |  |  |  |  |  |  |  |  |
| *E. glauca × Microcitrus* sp. | 1 | nd | 33.4 |  | nd | nd | nd |  |  |  |  |  |  |  |
|  | 2 | nd | 33.0 |  | nd | nd |  |  |  |  |  |  |  |  |
|  | 3 | nd | 33.3 |  | F | |  |  |  |  |  |  |  |  |
|  | 4 | nd | 33.7 |  | F | |  |  |  |  |  |  |  |  |

^a^ Plant Las-challenged by grafting from which stems were removed to attempt rooting

^b^ Months after inoculation by grafting

^c^ Number of rooted stem cutting per plant with were evaluated for Las infection

^d^ Non-detected

^e^ Rooting failled

*All plants were dead at the moment of the experiment

**Supplementary Table 6** *'Candidatus* Liberibacter asiaticus' detection in new flushes from Oceanian citrus genotypes and ‘Tobias’sweet orange as scions onto Las-infected ‘Rangpur’ lime rootstocks, one month after drastic pruning of the canopy at 5 cm above the scion-rootstock union.

| **Plant** | **Oceanian citrus genotypes** |
| --- | --- |
|  | **New Flush after prunning** |
| *C. × sinensis* (L.) Osbeck ‘Tobias’ | |
| 1 | 32.0 |
| 2 | 29.0 |
| 3 | F^a^ |
| 4 | F |
| 5 | d |
| 6 | 28.6 |
| 7 | 24.9 |
| 8 | 23.0 |
| 9 | 23.2 |
| *M. warburgiana* (F.M. Bailey) Tanaka | |
| 1 | F |
| 2 | d^b^ |
| 3 | F |
| 4 | d |
| 5 | F |
| 6 | d |
| 7 | F |
| 8 | F |
| 9 | d |
| *M. papuana* hybrid | |
| 1 | d |
| 2 | nd^c^ |
| 3 | nd |
| 4 | nd |
| 5 | d |
| 6 | d |
| 7 | nd |
| 8 | d |
| *M. australis* hybrid | |
| 1 | d |
| 2 | nd |
| 3 | nd |
| 4 | nd |
| 5 | nd |
| 6 | nd |
| 7 | nd |
| 8 | d |
| 9 | nd |
| 10 | nd |
| *E. glauca* (Lindl.) Swingle | |
| 1 | nd |
| 2 | nd |
| 3 | nd |
| 4 | nd |
| 5 | nd |
| 6 | nd |
| 7 | nd |
| *Microcitrus* sp. hybrid | |
| 1 | nd |
| 2 | nd |
| 3 | nd |
| 4 | nd |
| 5 | nd |
| 6 | nd |
| 7 | nd |
| *E. glauca* x *Citrus* sp. hybrid | |
| 1 | nd |
| 2 | nd |
| 3 | nd |
| 4 | nd |
| 5 | nd |
| 6 | nd |
| 7 | nd |
| 8 | d |
| 9 | nd |
| 10 | nd |
| 11 | nd |
| 12 | nd |
| *E. glauca* x *Microcitrus* sp. hybrid | |
| 1 | F |
| 2 | F |
| 3 | F |
| 4 | F |
| 5 | F |
| 6 | F |
| 7 | nd |
| 8 | F |

^a^ Failed: no new flush obtained after pruning

^b^ Plant died and evaluation was not possible to be done

^c^ Non-detected.
